# Insight into the influence of Re and Cl on Ag catalysts in ethylene epoxidation†

**DOI:** 10.1039/d4cy00858h

**Published:** 2024-11-06

**Authors:** Claudia J. Keijzer, Pim T. Weide, Kristiaan H. Helfferich, Justyna Zieciak, Marco de Ridder, Remco Dalebout, Tracy L. Lohr, John R. Lockemeyer, Peter van den Brink, Petra E. de Jongh

**Affiliations:** a Materials Chemistry and Catalysis, Debye Institute for Nanomaterials Science, Utrecht University The Netherlands P.E.deJongh@uu.nl; b Shell Global Solutions International Amsterdam The Netherlands; c Shell Catalysts & Technologies Houston Texas USA; d Shell Global Solutions US Inc. Houston Texas USA

## Abstract

Commercial ethylene epoxidation catalysts consist of α-alumina supported Ag particles and usually contain a mixture of promoters. High selectivity catalysts typically include a small amount of rhenium species. We studied a series of Ag catalysts promoted with Re loadings up to 4 at% (Re/(Re + Ag)), which is intentionally higher than in optimized commercial catalysts to facilitate characterization and to amplify the influence on catalysis. Sequential impregnation brought Re and Ag in such close contact that they formed a new characterized phase of AgReO_4_. Chemisorption experiments showed that both ReO_*x*_ and AgReO_4_ species act as a reversible reservoir for O_2_. Ethylene epoxidation was performed without and with the industrially crucial ethyl chloride promoter in the feed. Without the chloride (Cl), the ethylene oxide selectivity increased when Re was present, whereas the combination of Re and Cl decreased the ethylene oxide selectivity at higher Re loadings. Systematic ethylene oxide isomerization experiments revealed that Re and Cl individually inhibit the isomerization on the Ag surface. However, Re and Cl combined increased the isomerization, which can be explained by the surface becoming overly electrophilic. This hence shows the importance of studying promoters both individually and combined.

## Abstract 2

Crystalline AgReO_4_ was formed in Re-promoted Ag catalysts. The effect of Re on catalysis and EO isomerization was tested without and with the industrially relevant ethyl chloride. EO isomerization was greatly affected by both Re and Cl.

## Graphical abstract

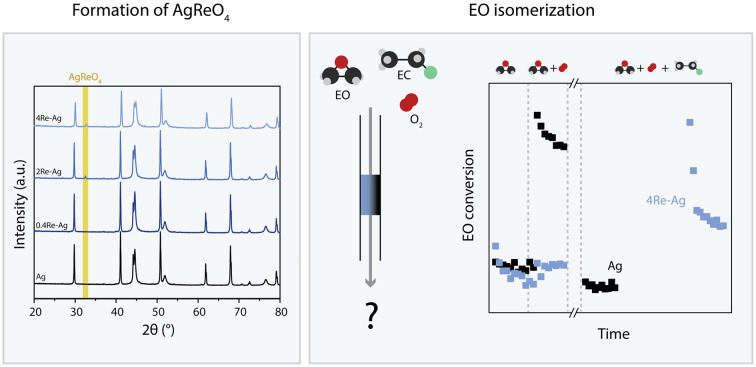

## 1. Introduction

Ethylene oxide (EO) is the primary building block for poly(ethylene glycol), which is used for making a myriad of chemicals such as antifreeze agents, insulating materials, and pharmaceuticals.^1–3^ EO is produced by epoxidation of ethylene, which is catalyzed by Ag particles on α-Al_2_O_3_.^4,5^ Ag displays unique activity and selectivity towards ethylene oxide compared to other metals due to its moderate oxidizing behavior.^6^ During ethylene epoxidation conditions, the Ag surface is partially oxidized with either electrophilic (weakly bound) or nucleophilic (strongly bound) oxygen.^7^ It is generally believed that electrophilic oxygen promotes the selective oxidation of ethylene to ethylene oxide, whereas nucleophilic oxygen results in total combustion.^8–10^ Ethylene oxide can isomerize to acetaldehyde, which is catalyzed by support hydroxyl groups, hence an inert support material such as α-alumina is used to suppress this side-reaction.^11^ Ag itself was also found to isomerize EO to acetaldehyde.^12^ Acetaldehyde subsequently oxidizes to CO_2_ and water on the Ag surface. The selectivity towards ethylene oxide depends on the ratio between the various reaction rates and the ratio between active surface and support sites.^11,13^

With CO_2_ as main side-product (0.2–0.3 Mt per Mt EO),^2^ it is of great importance to understand how the EO selectivity is influenced, and hence optimized. In industry, ethylene epoxidation runs at relatively low ethylene conversions (7–15%),^3^ since the EO selectivity inversely depends on the ethylene conversion due to subsequent side reactions.^14^ Another approach to increase selectivity is to introduce promoters to the catalyst. Gaseous organochlorides such as ethyl or vinyl chloride decompose to Cl species on the Ag surface, blocking oxygen vacant sites which suppresses the overall activity.^15^ In addition, Cl was found to increase the ratio between the concentration of electrophilic oxygen and nucleophilic oxygen on the Ag surface, which is beneficial for the selective oxidation pathway.^7^

Besides gaseous chloride compounds, a variety of solid promoters are typically used.^16,17^ In general, catalysts classified as “high activity” in industrial terminology contain alkali promoters and so-called “high selectivity” catalysts contain a mixture of rhenium and alkali species.^3^ The mechanism behind Re promotion in ethylene oxide catalysts has been a topic of debate,^18–22^ but literature addresses Re in combination with Cs and chloride,^18,19,22^ or only Re without the industrially used chloride promoter.^21^ In the latter, it was found that Re_2_O_7_ species block Ag sites with high oxygen affinities, inhibiting the formation of nucleophilic oxygen and increasing the EO selectivity.^21^ In another study with chloride in the feed, Re–Ag catalysts were found to be less active and less selective,^18^ while Re_2_O_7_ increased the amount of electrophilic oxygen. Dellamorte *et al.* described morphological changes within Ag catalysts with 25 ppm Re and mentioned the use of vinyl chloride in the feed.^23^ However, catalytic data and a clear understanding of the effect of Re is limited.

In addition, “high selectivity” catalysts contain low Re loadings (35–900 ppm of the total catalyst weight^5,17^). As a consequence, the effect of Re on (structural) properties of Ag catalysts is difficult to investigate. An intermediate [Ag(μ-ethylenediamine)][ReO_4_] phase, precipitated from a precursor solution, has been investigated in an earlier study which demonstrated that Re and Ag species can have a close proximity during the preparation of commercial catalysts.^20^ In this work, we study Ag catalysts with 0–4 at% Re loadings. Using X-ray diffraction (XRD), the formation of AgReO_4_ is studied, confirming the presence of Re^7+^ species, but not as the earlier believed Re_2_O_7_. Re-promoted Ag catalysts were tested without and with varying amounts of ethyl chloride in the feed to compare with literature. Re in absence of Cl inhibited the ethylene conversion and hence indirectly increased the EO selectivity, whereas Re and Cl together at high concentrations resulted in an overall decrease in selectivity. In addition to ethylene epoxidation experiments, we separately investigated EO isomerization behavior with and without chloride, which is crucial to understand EO selectivity.

## 2. Materials and methods

### 2.1 Catalyst preparation

Re-promoted Ag catalysts supported on α-alumina were prepared with sequential incipient wetness impregnation. Prior to the first impregnation, 8 m^2^ g^−1^ α-alumina extrudates (BASF, Al-4196, pore volume of 0.45 cm^3^ g^−1^) were crushed and sieved to a fraction <212 μm and dried in vacuum at 200 °C for 2 h. Ammonium perrhenate (NH_4_ReO_4_, ≥99%, Aldrich Chemistry and Fisher) was dissolved in MilliQ® water, obtaining solutions of 0.016, 0.08 and 0.16 M. The dried α-alumina was impregnated with one of these solutions up to 90% of its pore volume, aiming for final Re loadings of 0.4, 2 and 4 at% compared to the Ag loading (Re/(Ag + Re)). The impregnated material was dried in stagnant air at 60 °C for 20 h. After 15, 30 and 45 min of drying, all materials were mixed thoroughly to promote a homogeneous distribution. The dried samples were transferred to a U-shaped borosilicate reactor and calcined for 2 h at 500 °C (5 °C min^−1^ ramp) in 25% O_2_ in N_2_ (gas hourly space velocity (GHSV) of 7000 h^−1^).

The Ag deposition was based on a procedure described elsewhere.^24^ Silver oxalate was used as Ag precursor, which was synthesized by adding an aqueous silver nitrate (≥99.0%, Sigma-Aldrich) to an aqueous solution of oxalic acid (≥99.0%, Sigma-Aldrich) in a 2 : 1 mol ratio. Silver oxalate precipitated immediately and was washed three times in MilliQ® water and once in ethanol, after which it was dried in air at room temperature. Please note that silver oxalate is shock-sensitive, and should therefore be handled with great care.

For the deposition of Ag, the (Re-promoted) α-alumina samples were dried in vacuum at 200 °C for 2 h. Silver oxalate was dissolved in a MilliQ®/ethylene diamine (99%, Sigma-Aldrich) solution (4 : 1 mol ratio). The dried powder was impregnated up to 90% of its pore volume with the Ag_2_C_2_O_4_/MilliQ®/ethylene diamine solution, aiming for a Ag loading of 15 wt%. The pore volume of the Re-promoted α-alumina powder was assumed to be similar as the pure α-alumina. After impregnation, the material was subjected to a similar drying and calcination procedure as described for the Re-impregnation, except that the heating temperature was 215 °C.

AgReO_4_ was deposited on α-alumina *via* co-impregnation. Silver oxalate and ammonium perrhenate (Ag : Re mol ratio of 1 : 1) were dissolved in a MilliQ®/ethylene diamine solution (4 : 1 mol ratio). Dried α-alumina powder was impregnated with this solution, up to 90% of its pore volume, aiming for a AgReO_4_ loading of 15 wt%. A similar drying and calcination procedure as for the Ag deposition was performed after impregnation. Afterwards, a second heating step was performed for 2 h at 500 °C (5 °C min^−1^ ramp) in 25% O_2_ in N_2_ (GHSV of 7000 h^−1^).

### 2.2 Characterization

Scanning electron microscopy (SEM) was used to determine the Ag particle size of the samples. A FEI Helios G3 UC microscope was operated at 5–10 kV in immersion mode. Double-sided carbon adhesive tabs (Electron Microscopy Sciences) were used to attach the sample powder to the SEM holder, upon which a 7.5 nm PdPt layer was sputtered to create conductivity. For each sample, the diameter of at least 200 Ag particles was measured using ImageJ software. Surface averaged particle diameters (*d*_p,s_) with corresponding standard deviations (*σ*_p,s_) were calculated using eqn (1), with *n* the number of counted silver particles and *d*_*i*_ the diameter of particle *i*.1
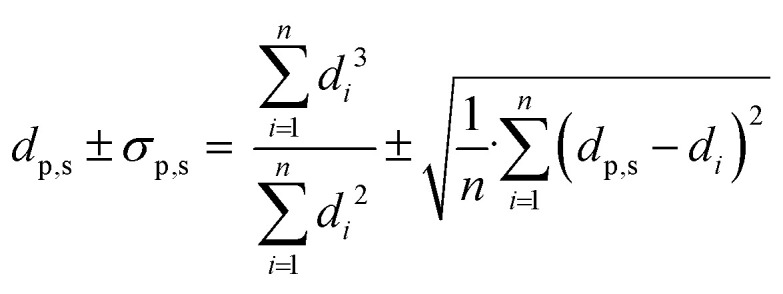
Scanning transmission electron microscopy with energy dispersive X-ray spectroscopy (STEM-EDX) was performed on a Talos F200x microscope equipped with a Super-X G1 EDX spectrometer, which was operated at 200 kV and 160 mm camera length. EDX maps were acquired using a spotsize of 6 and 2.5 gun lens, operating the microscope at *ca.* 300 nA with an acquisition time of 5 min. Identification of the EDX signal was performed using Velox™ software. A subtraction of the background EDX counts was performed using the net intensity. The image quality was increased by averaging the spectrum profiles per pixel over 3 × 3 pixels to enhance the possibility to detect low intensity features. Sample powders were dispersed in isopropanol and drop-casted on a holey carbon Cu grid (300 mesh).

Crystalline phases within the samples were determined with X-ray diffraction (XRD) using a Bruker D2 Phaser equipped with a Co K_α_ source (*λ* = 0.1789 nm) and operated at 30 kV and 10 mA, under constant rotation of 15 rpm. Diffractograms were measured between 20–80° 2*θ* with a step size of 0.020° 2*θ* and 1 s per step. The measured diffractograms were analyzed with Bruker TOPAS V5 software and fitted with theoretical diffractograms from the PDF-4+ 2016 database. The following PDF cards were used: 04-004-2852 (α-Al_2_O_3_), 04-003-5319 (Ag), 04-014-4906 (AgReO_4_) and 04-004-1280 (ReO_2_).^25^

O_2_ chemisorption was used to determine the O_2_ uptake of the catalysts, using a Micromeritics ASAP 2020 apparatus. 100–200 mg of sample was loaded in a U-shaped quartz reactor between two layers of quartz wool. The sample was evacuated at 100 °C for 30 min with a heating ramp of 10 °C min^−1^. Thereafter, the sample was flushed with O_2_ for 10 min at 100 °C, before increasing the temperature to 215 °C (10 °C min^−1^). This step was followed by an evacuation step and a treatment with H_2_ for 60 min, in order to clean the surface. After another evacuation step of 30 min, the O_2_ chemisorption experiment was performed with an equilibration time of 10 s.

X-ray photoelectron spectroscopy (XPS) analysis was conducted utilizing the advanced Nexsa G2 Surface Analysis System, featuring an X-ray photoelectron spectrometer equipped with a hemispherical energy analyzer and a monochromatic Al K_α_ source. Operating at 12 keV, the monochromatic Al K_α_ source underwent optimization, employing a pass energy of 250 eV for the survey scan and 50 eV for the subsequent high-resolution scan. The X-ray spot size was set at 400 μm during the analyses. In preparation for XPS measurements, all samples were processed into pressed powders and affixed to a stainless steel stub using 3 M 666 double-sided tape. A charge neutralizer was used to minimize charging of the sample surfaces. The spectra referenced to the Al 2p peak with a binding energy of 74.4 eV. Further details on the XPS interpretation are listed in section A of the ESI.†

Inductively coupled plasma (ICP) analysis was performed at Mikroanalytisches Laboratorium Kolbe to determine the weight loading of Re and Ag within the catalysts.

### 2.3 Ethylene epoxidation and ethylene oxide isomerization experiments

Prior to testing the catalysts in the ethylene epoxidation reaction, the catalysts were sieved to a 90–150 μm sieve fraction. In a typical catalytic run, 100 mg catalyst was diluted with 500 mg SiC (212–425 μm), which had been washed in nitric acid (65%, AnalaR Normapur®, 10 mL g_SiC_^−1^) and calcined at 800 °C to remove organic and inorganic impurities. The diluted catalyst was loaded in a quartz reactor (4 mm internal diameter) between two layers of quartz wool. The experiments were performed at 215 °C, with 7.5 vol% ethylene, 7.5 vol% oxygen, and ethyl chloride concentrations of 0–3 ppm in balance helium, with total gas flows ranging from 16–66 mL min^−1^. A fixed ratio was chosen, not optimizing the process conditions for each experiment, as the exact ratio is not critical for the selectivity as shown earlier.^26^ Reaction products were analyzed with an online Interscience Compact GC, equipped with a Porabond Q column and Molsieve 5 Å column. At room temperature, the reactant concentrations in the feed were evaluated to guarantee the feed composition.

Ethylene conversion and ethylene oxide selectivity were determined using eqn (2) and (3), respectively. Average conversions and selectivities were calculated after the catalysts had equilibrated at each EC concentration for 15–20 h. The carbon mass balance was calculated using eqn (4), and was 100 ± 3% for each datapoint.2
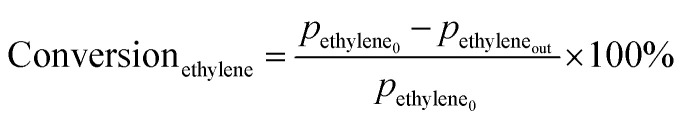
3
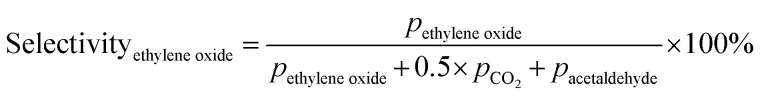
4
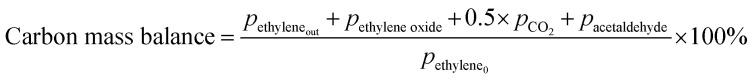
For ethylene oxide isomerization experiments, 100 mg of catalyst (90–150 μm) was loaded in a quartz reactor (4 mm internal diameter) between two layers of quartz wool. Isomerization tests were performed (without heat treatment or equilibration time) at 215 °C with 33 mL min^−1^ total gas flow of 0.15 vol% ethylene oxide, 0.075 vol% acetaldehyde (Linde HiQ), and 7.5 vol% oxygen in balance helium. To evaluate the influence of ethyl chloride (EC) on ethylene oxide isomerization, catalysts were first tested in the ethylene epoxidation reaction and stabilized with EC for *ca.* 50 h, prior to isomerization experiments with 1 ppm EC. Ethylene oxide conversion was calculated using eqn (5).5
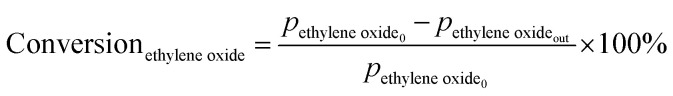
Selectivities towards isomerization products (acetaldehyde, CO_2_ and ethylene) were calculated with eqn (6)–(8). The outlet acetaldehyde was corrected for the amount of acetaldehyde in the reactant gas mixture.6
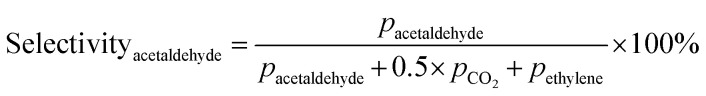
7
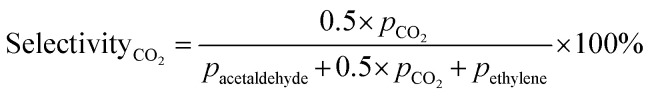
8
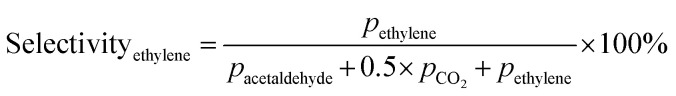
Stacked-bed experiments were performed at 295 °C with 20 mg γ-Al_2_O_3_ (Engelhard, 250 m^2^ g^−1^, 90–150 μm) positioned on top of 100 mg catalyst, with similar concentrations as for the isomerization experiments.

## 3. Results and discussion

### 3.1 Catalyst structure

**Fig. 1:**
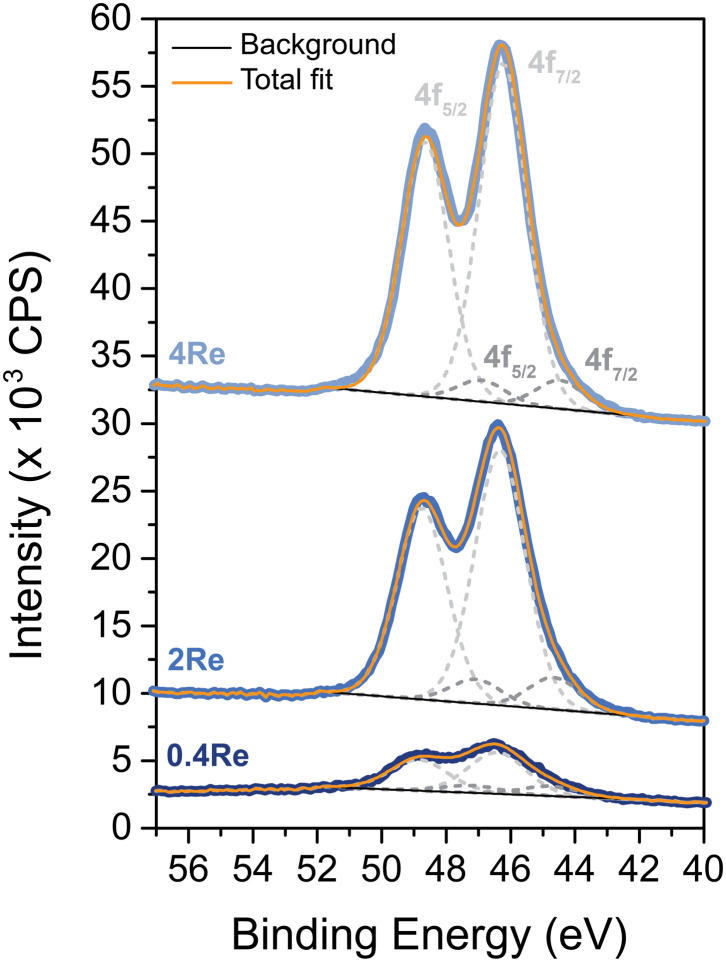
X-ray photoelectron spectra of the Re 4f region of re on α-alumina after calcination at 500 °C. Sample names refer to the final Re loading after Ag deposition in at% (Re/(Re + Ag)). Light grey fits correspond to Re^7+^ and dark grey fits correspond to Re^6+^.

The oxidation state of Re on α-alumina prior to Ag deposition was determined with XPS, as no crystalline ReO_*x*_ phases within the 4Re sample could be detected with XRD (Fig. S3†). Rhenium can have several oxidation states varying from −3 to +7.^27^Fig. 1 shows the spectra of Re-promoted α-alumina. The sample names refer to the final Re loading after silver deposition in at% (Re/(Re + Ag)). For all samples, the most dominant Re 4f_5/2_ and 4f_7/2_ peaks are around 49 and 46 eV, which is typical for an oxidation state of +7. A minor mismatch between the Re^7+^ fits and the spectra at lower binding energies suggests the presence of Re^6+^, with 4f_5/2_ and 4f_7/2_ peaks around 47 and 44 eV, respectively. Zoomed-in spectra are shown in Fig. S1.† The Re^6+^/Re^7+^ ratio increases from 0.08 to 0.23 when the Re loading decreases from 4 to 0.4 at% (Table S1†), which might be explained by an increasing surface-to-bulk ratio. Nevertheless, Re^7+^ remains the predominant phase in these samples. Re^7+^ corresponds to the Re_2_O_7_ stoichiometry, which is also reported in literature after calcination of ReO_*x*_ above 500 °C.^28^

**Fig. 2:**
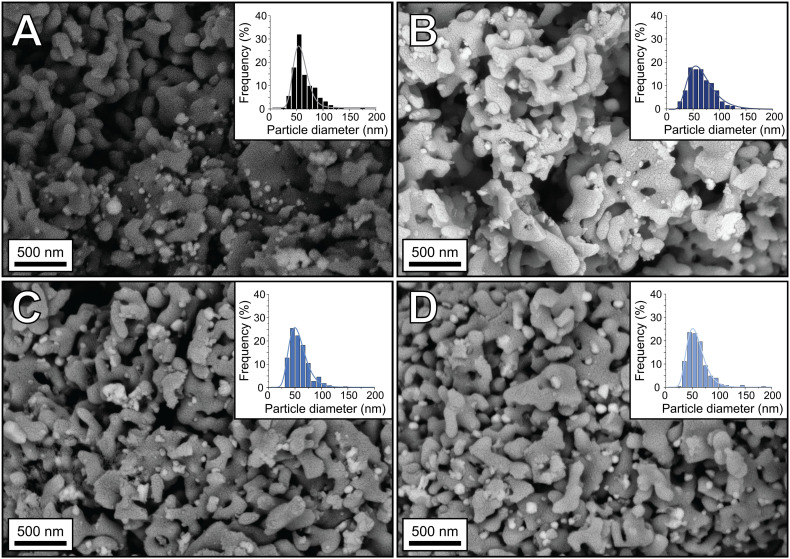
SEM images of Ag (A) and Re-promoted Ag catalysts, with 0.4 at% Re (B), 2 at% Re (C), and 4 at% Re (D). Histograms of particle diameters are shown as insets. For each catalyst more than 200 Ag particles were measured.

Once Ag particles were deposited on the (Re-promoted) α-alumina, SEM was performed. Fig. 2 shows the alumina-supported silver catalysts with rhenium loadings of 0 to 4 at% compared to silver. The silver particles are depicted as white to light grey spheres on the darker grey α-alumina support. Re at such low loadings could not be distinguished from the Ag. The surface averaged particle diameters of the (Re-promoted) Ag catalysts are summarized in Table 1, together with the theoretical silver and rhenium loadings and the experimental loadings measured with ICP and XPS. The particle diameters of all catalysts are 70–80 nm, but increasing the Re loading (above 0.4Re–Ag) slightly narrowed the particle diameter distributions. In literature, Re was reported to cause a trimodal Ag particle size distribution, or to have no effect on particle size at all.^18,22,23^ In our present study, relatively high loadings of Re were deposited prior to the Ag, which might have increased the concentration of anchoring sites for the Ag precursor. Since the Ag loading and particle sizes are similar, differences in catalyst performance can be ascribed to the influence of Re rather than to Ag particle size effects or a different surface ratio between Ag and α-alumina.^13^ The experimental loadings determined with ICP were as expected, whereas the Re loading determined with XPS was higher than the ICP measurements. As XPS is a surface sensitive technique, this means that the Re is preferentially located at the surface rather than in the bulk of the material.

**Table 1:** Overview of (Re-promoted) silver catalysts. Ag surface averaged particle diameters (*d*_p,s_) were determined with SEM. Theoretical Ag and Re loadings are compared with ICP and XPS data. Catalysts are labelled as [at%]Re–Ag

Catalyst	Ag particle diameter *d*_p,s_ (nm)	Ag loading (wt%)		Re/(Re + Ag) (at%)		
		Theoretical	ICP	Theoretical	Bulk, ICP	Surface, XPS
Ag	79 ± 26	14.8	14.5	0	0	0
0.4Re–Ag	80 ± 27	14.6	14.6	0.39	0.37	3.07
2Re–Ag	71 ± 23	15.9	14.8	2.08	2.22	9.67
4Re–Ag	72 ± 15	14.7	14.3	3.87	3.55	15.27

**Fig. 3:**
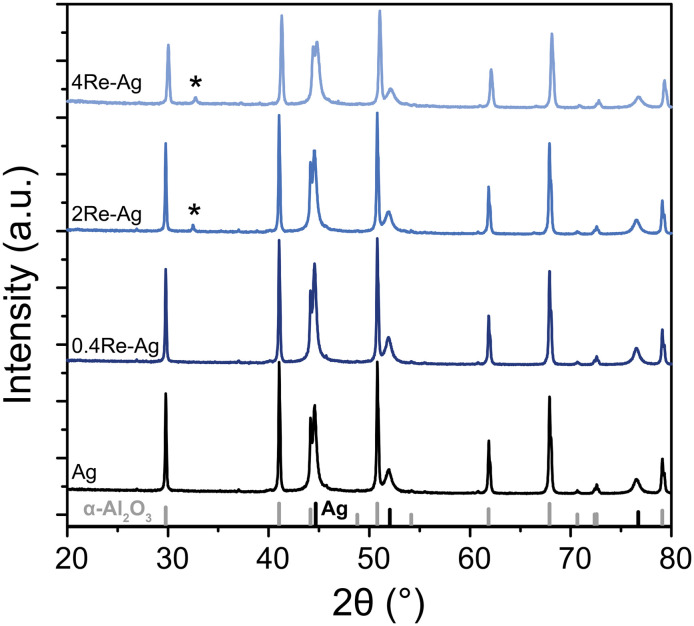
X-ray diffractograms of the Ag and Re-promoted Ag catalysts. Theoretical stick diffraction patterns of α-Al_2_O_3_ (grey) and Ag (black) are depicted below the diffractograms as reference. The asterisk (*) indicates AgReO_4_.

To determine the crystalline phases in the samples, X-ray diffraction was performed. Fig. 3 shows the diffractograms of the unpromoted and Re-promoted Ag catalysts, with reference diffractograms of α-Al_2_O_3_ and Ag at the bottom of the Figure. The diffractograms of the Ag and 0.4Re–Ag catalysts only show Ag and α-Al_2_O_3_ peaks. For 2Re–Ag and 4Re–Ag, however, a small diffraction line around 32° 2*θ* was detected, which belongs to AgReO_4_ which is a thermodynamically stable phase under these conditions (Fig. S14†). Previously, an intermediate phase [Ag(μ-ethylenediamine)][ReO_4_] had been identified during the preparation of ethylene epoxidation catalysts,^20^ where Ag and Re precursors were dissolved in the same solution. In another study, AgReO_4_ colloids were synthesized in solution.^29^ We show that also sequential impregnation can bring Re and Ag in such close contact that they form a single crystalline phase upon calcination.

The Re in the samples containing Ag (and thus AgReO_4_) has a predominant oxidation state of +7 according to XPS (Fig. S1 and Table S1†). In literature, a similar oxidation state has been reported, but it was attributed to Re_2_O_7_ rather than to AgReO_4_.^18^ Prolonging the calcination time from 2 to 12 h increased the amount of crystalline AgReO_4_ from 0.5 to 0.8 wt% in the sample (Fig. S4 and Table S3†). Re_2_O_7_ has a relatively low melting temperature of 297 °C,^30^ which might explain its mobility on the α-Al_2_O_3_ support and contact with the Ag surface during calcination. In patent literature, Re-promoted Ag catalysts are conditioned for several hours in an O_2_-containing feed without ethylene.^31,32^ It is hence expected that AgReO_4_ species also form in commercial catalysts with less Re, although in a lower concentration and hence more difficult to detect.

**Fig. 4:**
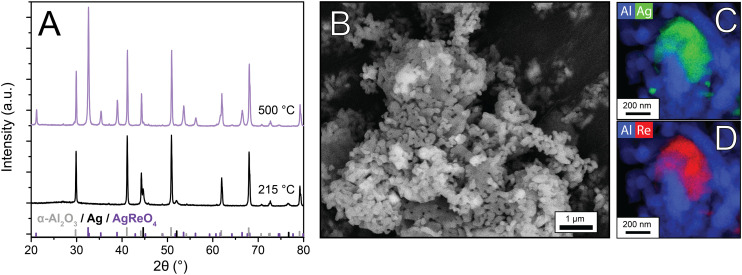
X-ray diffractograms of the AgReO_4_ samples calcined at 215 and 500 °C, with theoretical stick diffraction patterns of α-Al_2_O_3_, Ag and AgReO_4_ below (A), SEM image of AgReO_4_/α-Al_2_O_3_ calcined at 500 °C (B) and STEM-EDX maps of a zoomed in AgReO_4_/α-Al_2_O_3_ particle (C and D).

Industrial Ag catalysts contain low Re loadings (35–900 ppm of the total catalyst weight^5,17^), and consequently the influence of Re on structural properties is difficult to investigate. By increasing the Re loading, characterization becomes feasible, and effects on catalysis are amplified, which can give insight into the working mechanism of Re as a promoter. Therefore, once AgReO_4_ was detected in the Re-promoted Ag catalysts, 15 wt% AgReO_4_ was deposited on α-alumina to further investigate the properties of this phase. After calcination treatments at 215 °C and 500 °C, X-ray diffractograms, SEM, and STEM-EDX images were collected (Fig. 4). At 215 °C no crystalline AgReO_4_ had formed, whereas at 500 °C intense diffraction lines due to AgReO_4_ are visible. Calculated crystallite sizes are *ca.* 70 nm, which is also in the upper limit of the XRD due to experimental line broadening, whereas the AgReO_4_ particles visible in SEM are 100–500 nm and are shaped irregularly (Fig. 4B) which implies that these particles contain multiple crystalline domains. STEM-EDX maps of Al–Re (Fig. 4C) and Al–Ag (Fig. 4D) confirm that Ag and Re are in close proximity, but also small Ag particles are present on the α-Al_2_O_3_ support. In contrast, XRD does not show crystalline Ag peaks, possibly due to the detection limit, which suggests that the bulk is mostly AgReO_4_. AgReO_4_ colloids have been reported earlier,^29^ but to our knowledge have never been deposited on a support material.

### 3.2 Effect of Re on O_2_ uptake

During ethylene epoxidation, both the adsorption of oxygen on the silver surface and the subsequent reaction of ethylene with the adsorbed oxygen can be rate-limiting steps.^33,34^ Hence the adsorption of oxygen is an important parameter to consider.^35^ Re is a redox-active element with oxidation states between −3 to +7,^27^ and in the case of pure rhenium oxides this corresponds to an oxygen/rhenium ratio varying between 0 and 3.5. The effect of Re on O_2_ uptake was investigated using O_2_ chemisorption at 215 °C, which is the same temperature used for catalytic testing. Table 2 summarizes the O_2_ uptake of the (Re-promoted) catalysts, prior to and after Ag deposition. It should be noted that the samples were reduced at 215 °C in H_2_ for 60 min prior to the O_2_ chemisorption analysis to clean the surface, following a protocol developed to characterize the active silver surface.^36^ O_2_ isotherms are shown in Fig. S6 and S7.† For comparison, the fraction of crystalline AgReO_4_ in the samples determined with XRD is also listed.

**Table 2:** XRD results together with O_2_ chemisorption data performed at 215 °C of the Ag and Re-promoted Ag catalysts

Sample	Crystalline AgReO_4_^a^ (wt%)	Total O_2_ uptake (μmol_O_2__ g_sample_^−1^)	Total O_2_ uptake – O_2_ uptake by Ag^b^ (μmol_O_2__ g_sample_^−1^)
2Re	0	18.6	18.6
4Re	0	37.6	37.6
Ag	0	16.3	0
0.4Re–Ag	0	17.0	0.7
2Re–Ag	0.28 ± 0.02	45.5	29.2
4Re–Ag	0.48 ± 0.03	74.5	58.2
AgReO_4_	10.78 ± 0.05	171.8	171.8

a Crystalline fraction of the sample, determined with XRD after 2 h calcination at 215 °C except for sample AgReO_4_ which was subsequently calcined at 500 °C.

b The O_2_ uptake of Ag was subtracted from the total O_2_ uptake of the Re–Ag catalysts, assuming the O_2_ uptake by Ag was similar in these catalysts. For the Re and AgReO_4_ samples no crystalline Ag was present, hence no subtraction was performed.

The 2Re and 4Re samples both contain Re_2_O_7_ and show significant O_2_ uptake, which can be explained by the reoxidation of the Re-containing samples after the treatment in H_2_ to clean the surface. In case of 2Re–Ag and 4Re–Ag, the O_2_ uptake is higher than of the individual Ag and Re samples combined. The formation and/or presence of AgReO_4_ in these samples might explain the increased O_2_ uptake. 0.4Re–Ag has a similar uptake as the Ag sample (Fig. S7†) and no detectable AgReO_4_ with XRD. The AgReO_4_ sample showed the highest O_2_ uptake of 172 μmol_O_2__ g_sample_^−1^ with a corresponding 10.78 wt% crystalline AgReO_4_. It is clear from these experiments that both ReO_*x*_ and AgReO_4_ species are a reversible reservoir for O.

Repeating the measurement on the AgReO_4_ sample resulted in a lower O_2_ uptake of 107 μmol g_sample_^−1^ (Fig. S8†). During the chemisorption experiments the samples are treated in H_2_ at 215 °C to clean the surface, and according to XRD this resulted in a decrease of crystalline AgReO_4_ from 10.8 to 6.6 wt% and the formation of 1.3 wt% Ag (Fig. S6 and Table S3†), which explains the lower O_2_ uptake. XPS measurements were conducted after subjecting the AgReO_4_, 4Re–Ag and 4Re samples to similar gas treatments used for the chemisorption experiments. After reduction, the Re was reduced, containing Re in oxidation states down to 0, but after re-oxidation almost all Re^7+^ was regained for the AgReO_4_ and 4Re–Ag samples (Fig. S2 and Table S2†). AgReO_4_ shows interesting behavior in these reduction and oxidation cycles, which might be relevant for other reactions that require redox-active sites, and for the role of Re as a promoter for the Ag-catalyzed ethylene epoxidation.

### 3.3 Ethylene epoxidation

**Fig. 5:**
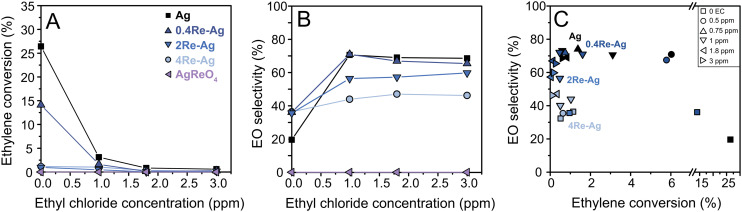
Ethylene conversion (A) and EO selectivity (B) as a function of the ethyl chloride concentration, at 215 °C and in a total gas flow of 66 mL min^−1^. (C) EO selectivity as a function of ethylene conversion at different EC concentrations, assigned with different symbols.

The Re-promoted Ag catalysts were tested in the ethylene epoxidation reaction. In this reaction, a chloride promotor is typically co-fed in the reactant gas feed to increase the ethylene oxide selectivity by modifying the nature of the active silver sites.^3,7^ Depending on the composition of solid promotors (alkali only or rhenium with alkali), different chloride concentrations show optimal activity and selectivity results, which is often not taken into account in academic studies but essential to compare different promoter compositions.^3^ Therefore, the catalysts were evaluated with varying chloride levels. Fig. 5A shows the ethylene conversion *versus* ethyl chloride concentration. The effect of rhenium is most pronounced without ethyl chloride, which is in line with literature:^37^ the conversion decreases with an order of magnitude upon increasing Re, and AgReO_4_ does not show a significant catalytic activity. Raw catalytic data are shown in Fig. S9.† In literature, Ag catalysts promoted with small amounts of Re (and no Cl) also have a decreased activity compared to Ag-only catalysts.^21^ Possibly, certain Ag sites are blocked by dispersed [ReO_4_]^−^ species. In the AgReO_4_ sample with crystalline AgReO_4_ it might be that no metallic Ag remains due to the 1 : 1 ratio between Ag and Re. The crystalline AgReO_4_ loading is unaltered after catalysis with EC (Table S3†), which shows that this phase is stable in ethylene epoxidation reaction conditions. The particle diameters for all Re-promoted Ag catalysts increased from 70–80 nm to approximately 90 nm after ethylene epoxidation (Fig. S18†), which implies that Re promotion did not significantly affect the stability of the Ag particles.

Ethylene conversion decreases for all catalysts upon increasing the chloride concentration. In literature, catalysts promoted with both Re and Cs are reported to show an increase in activity with increasing chloride concentrations.^3^ The origin of this increase in activity with both Cs and Re is still under debate: it is speculated that [ReO_4_]^−^ serves as molecular spacer for Cs-species and thereby preventing the formation of CsCl.^38^ The Re loadings in our study are intentionally higher than in industrial catalysts to study the influence of Re and Cl on ethylene epoxidation, without Cs. Catalytic tests between 0–1 ppm ethyl chloride (EC) were performed to investigate if the conversion would show an increase within this lower EC range (Fig. S9–S11†). The Re–Ag catalysts did not show an increase in conversion between 0–1 ppm EC (Fig. S12†), and hence with such high Re loadings and without Cs behave differently than the earlier reported Re–Cs promoted Ag catalysts.

Fig. 5B shows the EO selectivity as a function of EC concentration. Rhenium increases the ethylene oxide selectivity at 0 ppm EC (albeit, at a lower conversion). For ethylene epoxidation, a decrease in conversion typically causes an increase in selectivity due to the limitation of secondary reactions.^24^ This effect is illustrated in Fig. 5C which displays EO selectivity *vs.* conversion plots of the catalysts. Interestingly, without the chloride the Re-promoted catalysts show similar selectivities of *ca.* 35%, while 0.4Re–Ag shows a much higher conversion compared to 2Re–Ag and 4Re–Ag. Upon increasing the chloride concentration the Ag and 0.4Re–Ag catalysts show similar selectivities at relatively similar conversions, which is in line with literature,^37^ but the selectivity decreases with increasing Re loading (Fig. 5C). In this research, we deliberately chose higher Re loadings compared to the optimized Re loadings used in industry (35–900 ppm of the total catalyst weight),^5,17^ and 0.4Re–Ag is the only catalyst that reaches the upper limit of these low Re loadings (*ca.* 900 ppm). Therefore, it should be noted that the influence of Re on catalysis is intentionally magnified to provide insights into the mechanism of Re promoted silver catalysts, but does not represent the optimum composition for effective catalysts.

### 3.4 Ethylene oxide isomerization as measure for total selectivity

**Fig. 6:**
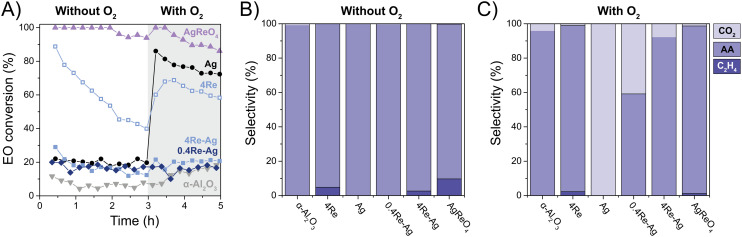
Ethylene oxide (EO) conversion without and with oxygen (A), and corresponding product selectivities without (B) and with oxygen (C). Experiments were performed with 0.15 vol% ethylene oxide (EO), 0.075 vol% acetaldehyde (AA), 7.5 vol% O_2_ in He at 215 °C. No ethyl chloride has been added in these experiments.

EO isomerization is an important factor influencing EO selectivity.^11,14^ Inert supports limit isomerization, but EO can also isomerize and combust on the Ag surface.^12^ We performed dedicated EO isomerization experiments to understand the origin of the changes in EO selectivity during ethylene epoxidation by Re and EC. Fig. 6 shows the EO isomerization data without O_2_, and additionally with O_2_ in the feed to approach catalytic reaction conditions at 0 ppm EC. Without oxygen the Ag, 0.4Re–Ag and 4Re–Ag catalysts have similar EO conversions, whereas 4Re and AgReO_4_ show remarkably high EO conversions of 50 to 100%, respectively. Re_2_O_7_ is a Lewis acid,^39^ which might explain the high isomerization activity of 4Re. Calculations of equilibrium concentrations show that AgReO_4_ and Re_2_O_7_ reduce to Ag, ReO_2_ and ReO_3_ during EO isomerization without O_2_, but are thermodynamically stable in the presence of O_2_ (Fig. S14†). XRD indeed showed that the amount of crystalline AgReO_4_ in the AgReO_4_ sample decreased from *ca.* 10 to 5 wt% during EO isomerization and that Ag and ReO_2_ species formed (Fig. S6 and Table S3†).

Strikingly, introducing O_2_ in the feed did not change the EO conversions of the 0.4Re–Ag and 4Re–Ag catalysts. As these conversions are similar to the α-Al_2_O_3_ (*ca.* 20%), this suggests that the only sites active in EO isomerization are the support surface groups. Moreover, it seems that no separate ReO_*x*_ species are present in the 0.4Re–Ag and 4Re–Ag samples, as these would have increased the EO conversion as shown for the 4Re sample. In contrast, the unpromoted Ag catalyst now has an EO conversion of *ca.* 70%. These EO conversion trends are inversely related with the selectivity trends from Fig. 5 at 0 ppm EC, where 0.4Re–Ag and 4Re–g have similarly increased EO selectivities compared to the Ag catalyst (albeit, at lower conversions). A small amount of Re thus inhibits all EO isomerization on the Ag surface without chloride in the feed, which shows the value of Re in commercial catalysts.

Product selectivities during EO isomerization experiments give further insight into the mechanism of Re-promoted Ag catalysts. Without O_2_ in the feed, mostly acetaldehyde is formed (Fig. 6B), which is the direct product of isomerization and its formation does not require oxygen. Strikingly, ethylene was detected during tests with 4Re, 4Re–Ag and AgReO_4_. Stacked-bed experiments confirmed that ethylene was formed from ethylene oxide and not from acetaldehyde in the gas feed (Fig. S15†). It is known that Re^7+^ species catalyze the deoxygenation of epoxides to alkenes.^40^ Trace amounts of O_2_ and CO_2_ were detected in the feed (Fig. S16†). In the case of the Ag catalyst this might be caused by the release of weakly adsorbed O_2_, resulting in EO combustion. 4Re, 4Re–Ag and AgReO_4_ form slightly more O_2_, but these samples also display high O_2_ uptakes at 215 °C as determined with O_2_ chemisorption (Table 2), which underestimates the release of O_2_ upon ethylene formation.

With oxygen in the feed (Fig. 6C), CO_2_ is formed by most of the catalysts, which is the result of the total oxidation of acetaldehyde and/or EO. Compared to the isomerization without oxygen, less ethylene is formed for the 4Re and AgReO_4_ samples. When increasing the Re loading from 0.4 to 4 at% the CO_2_ selectivity decreases drastically to 8%. Not only does a small amount of Re inhibit the EO conversion to acetaldehyde, but it also inhibits acetaldehyde (or EO) combustion on the Ag surface and decreases the amount of CO_2_ emitted.

**Fig. 7:**
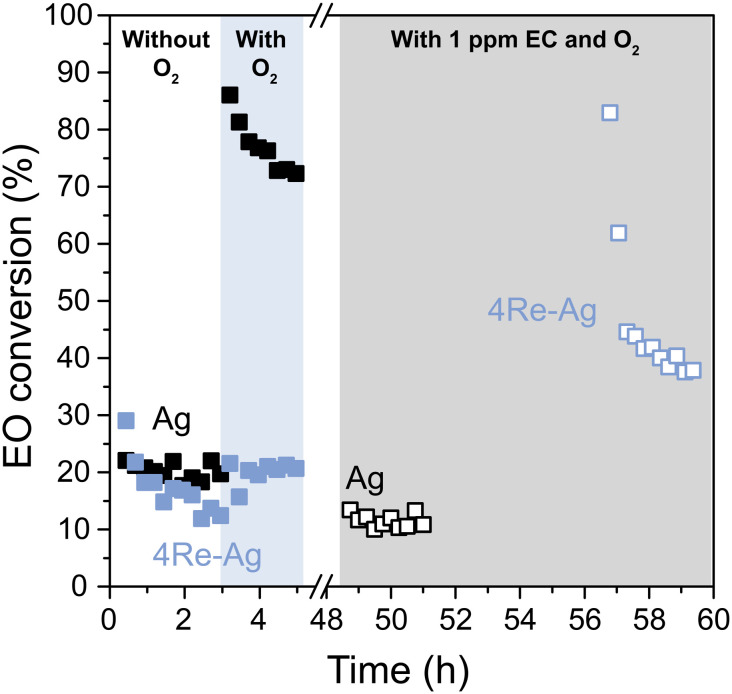
EO isomerization activity of the Ag catalyst (black) and 4Re–Ag catalyst (blue), without (closed symbols) and with ethyl chloride (open symbols) in the feed after ethylene epoxidation had taken place for *ca.* 50 h at 215 °C.

The EO isomerization results are in line with the selectivity trends without ethyl chloride (EC) in the feed. With EC, the ethylene oxide selectivity decreases with increased Re loading (Fig. 5). As EC is an industrially used promotor, EO isomerization tests were also performed after stabilizing the Ag and 4Re–Ag catalysts with the chloride during catalysis (Fig. 7). In earlier EO isomerization studies no ethyl chloride was co-fed and only (alkali promoted) Ag catalysts or supports were investigated.^41–43^ Interestingly, EC decreases the EO conversion of the Ag catalyst from 75 to 10%. We tested another Ag catalyst supported on α-alumina from a different batch, which displayed similarly low EO conversions after stabilization with EC (Fig. S17†). This contradicts an earlier study on Cl_2_-promoted Ag(111) crystals where Cl_2_ was reported to promote EO isomerization.^44^ It is unclear, however, whether in these previous studies these Ag(111) crystals were stabilized with Cl_2_ during ethylene epoxidation for a prolonged period, which is required for a meaningful evaluation of the catalysts' performance and can take up at least 10–20 h.^3,13^ To our knowledge, this is the first time that (Re-promoted) Ag catalysts have been systematically studied under industrially relevant isomerization conditions including the gaseous chloride promoter.

During ethylene epoxidation, the Ag surface is partially oxidized with either electrophilic or nucleophilic oxygen.^7^ Electrophilic oxygen promotes the formation of ethylene oxide, whereas nucleophilic oxygen results in total combustion.^8–10^ EC is known to increase the concentration of electrophilic oxygen species on the silver surface and decrease the nucleophilic oxygen.^7^ As nucleophilic oxygen is more prone to attack the C–H bond of EO and hence to catalyze its combustion, a reduction in nucleophilic oxygen species explains a decrease in EO conversion. In contrast, the 4Re–Ag catalyst shows an increased EO conversion, which is in line with the decreased EO selectivity from Fig. 5 with EC. Possibly, electrophilic O species are promoted by the chloride, while a surplus of AgReO_4_ make the O species overly electrophilic. This can activate the C–O bond from ethylene oxide which hence increases the EO conversion. It is clear that by studying AgReO_4_ separately, it is not merely a spectator phase but influences pathways of the ethylene epoxidation mechanism. In addition, we show that for both the unmodified and EC-modified experiments, the degree of EO isomerization accounts for the difference in EO selectivity for all catalysts which emphasizes the importance of separately understanding isomerization behavior and investigating catalysts after stabilization with the chloride.

## 4. Conclusions

Crystalline AgReO_4_ formed in Re–Ag catalysts, which has not been identified before. Previously reported Re^7+^ species likely originate from this AgReO_4_ phase, rather than from separate Re_2_O_7_ species, as was assumed so far. Both ReO_*x*_ and AgReO_4_ species are reservoirs for O_2_. The catalysts were tested without and with the industrially relevant ethyl chloride promoter in the feed and in all cases, Re decreased the activity due to [ReO_4_]^−^ blocking active silver sites. Without chloride, Re increased the EO selectivity while lowering the conversion. The combination of chloride and Re resulted in similar conversions and selectivities for 0.4Re–Ag and Ag, but the 2Re–Ag and 4Re–Ag showed a decreased EO selectivity compared to Ag at equivalent conversions. Including the industrially relevant ethyl chloride, we show for the first time that these changes in selectivity relate to the catalysts' EO isomerization behavior. This shows that the effects of chloride and Re are not limited to the selective oxidation pathway, but should also be considered for the subsequent isomerization reaction which influences the EO selectivity.

## Data availability

The data supporting this article have been included as part of the ESI.†

## Conflicts of interest

There are no conflicts to declare.

## Supplementary Material

CY-015-D4CY00858H-s001
