# Supplementary material for: Insight into the influence of Re and Cl on Ag catalysts in ethylene epoxidation
Source: Catal Sci Technol. 2024 Nov 6;15(2):323–33. doi: 10.1039/d4cy00858h (PMC11563198; doi:10.1039/d4cy00858h)
Supplement: CY-015-D4CY00858H-s001 [file CY-015-D4CY00858H-s001.pdf]

Supplementary Information (SI) for Catalysis Science & Technology.
This journal is © The Royal Society of Chemistry 2024

Supporting Information for 'Insight into the Influence of Re and Cl on Ag Catalysts in Ethylene Epoxidation'

Section A. XPS data 2

Section B. Additional XRD data 6

Section C. O_2 chemisorption isotherms 11

Section D. Additional catalytic data 13

Section E. Calculated equilibrium concentrations 16

Section F. EO isomerization and stacked bed studies 18

Section G. Additional SEM data of used catalysts 21

Section A. XPS data

Figure S1 shows X-ray photoemission spectra of Re/α-Al_2O_3 samples and Ag-Re/α-Al_2O_3 samples. XP spectra were fitted with CasaXPS (version 2.3.23) by a non-linear least-squares fitting algorithm using mixed Gaussian-Lorentzian (30/70) curves after linear background subtraction. The binding energy was calibrated using the Al 2p peak at 74.4 eV as a reference. Re $4f_{5/2}$ and $4f_{7/2}$ peaks were fitted with a fixed energy difference of 2.43 eV and a fixed $4f_{5/2}/4f_{7/2}$ peak area ratio of 0.75. A linear background was used instead of a Shirley type background, since there were also contributions of the Ag 4p peak and the Ca 3s peak, and possibly a loss peak of the O 2s. These contributions would mostly affect the Re 4f peaks of the 0.4Re and 0.4Re-Ag samples.

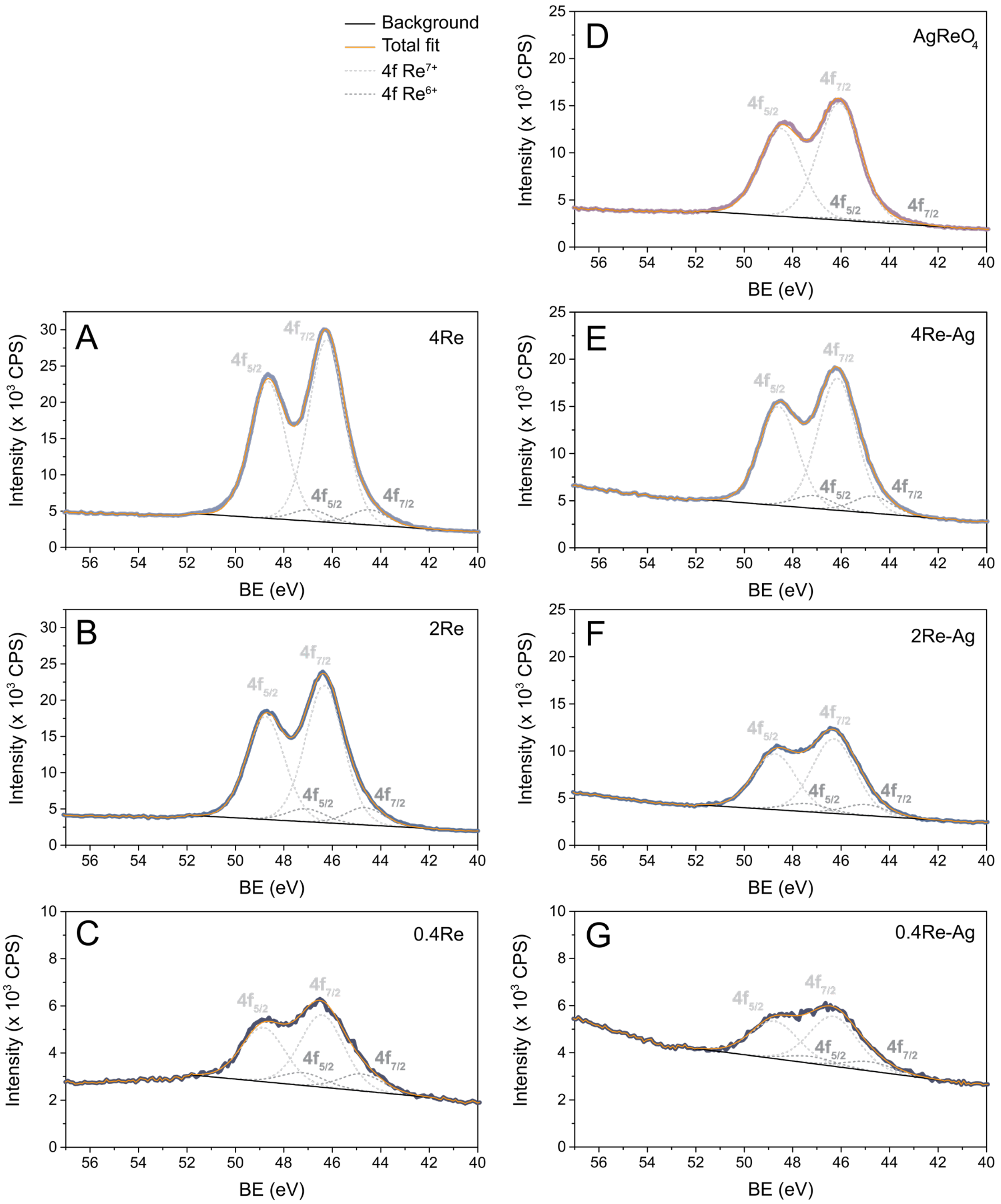

Figure S1. X-ray photoelectron spectra of the Re 4f region of Re on α-alumina (A-C) and Re-Ag on α-Al_2O_3 samples (D-G). Backgrounds are in black, Gaussian-Lorentzian fits of the 4f5/2 and 4f7/2 peaks are depicted in light grey (Re^{7+}) and dark grey (Re^{6+}), and the total fits are depicted in orange.

The ratio between Re^{6+}/Re^{7+} was calculated using the combined $4f_{5/2}$ and $4f_{7/2}$ peak areas of both components, and is listed for each sample in Table S1.

Table S1. Re 4f ratios between Re^{6+} and Re^{7+} determined using the total peak areas of each component.

**Sample**	**Re^{6+}/Re^{7+} ratio**
**$AgReO_4$**	0.02
**4Re**	0.08
**4Re-Ag**	0.13
**2Re**	0.13
**2Re-Ag**	0.15
**0.4Re**	0.23
**0.4Re-Ag**	0.18

In addition, the effect of reductive and oxidative atmospheres during the chemisorption experiments on the $AgReO_4$, 4Re and 4Re-Ag samples was investigated with X-ray photoelectron spectroscopy (XPS) using a Kratos Axis Supra+ apparatus, featuring an X-ray photoelectron spectrometer equipped with a hemispherical energy analyzer and a monochromatic Al K_α source. The survey spectra were recorded with a pass energy of 160 eV and the high resolution spectra with 20 eV. The X-ray spot size was set to 300-700 µm (slot) during the analyses. All samples were pressed into a quartz stub. Samples were treated under static conditions (technical air (20% O_2 in N_2) or 100% H_2) at 1-1.5 bar. First a cleaning step was done by an oxidative treatment at 215 °C in technical air, with a heating ramp of 5 °C min^{-1} and an isothermal step for 1h. This was followed by a reduction step at the same temperature in H_2 for 1h, after which a final oxidation step was performed at 215 °C in technical air for 4 h. Spectra were collected after the sample had cooled down (<100 °C) and was transferred to the analysis chamber (pressure ~7 x 10^{-12} bar) after each gas treatment. A charge neutralizer was used to minimize charging of the sample surfaces. The spectra were referenced to the Ag 3d peak with a binding energy of 368.3 eV. XP spectra of the $AgReO_4$ sample after the three gas treatments are shown in Figure S2. To resolve the Re 4f peak, a Shirley background was used and Gaussian-Lorentzian peak fitting.

After the first oxidation of the sample (Figure S2A), Re 4f consists of most likely two components: Re^{7+} and a lower oxidation state of rhenium, likely Re^{6+}. After reduction the spectrum still indicates the presence of Re^{7+} (Figure S2B). However, also peaks from lower oxidation states down to Re^0 appear. The spectral fits focussed on the determination of the Re^{7+} fraction and the presence of the Re^0

component. Chemical speciation of the other Re oxidation states has not been conducted. After re-oxidation, Re^{7+} becomes the most dominant phase again.

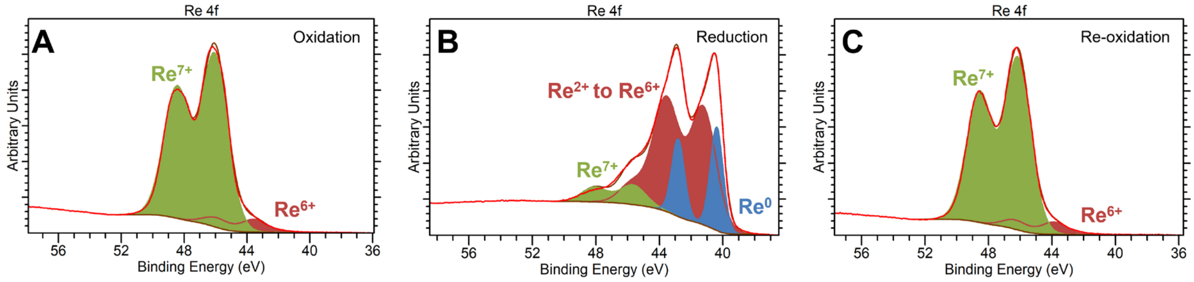

Figure S2. X-ray photoelectron spectra of the $AgReO_4$ sample after oxidation (A), reduction (B), and re-oxidation treatment (C) at 215 °C.

The relative fractions of each rhenium chemical state within the $AgReO_4$, 4Re-Ag and 4Re samples after the different treatments were calculated from deconvoluted peak area, which were corrected with a relative sensitivity factor, transmission function and inelastic mean free path (Table S2). It is clear from these experiments that the Re is reduced during the reduction treatment, but that the re-oxidation treatment recovers most of the Re^{7+}, especially for the $AgReO_4$ and 4Re-Ag samples which contain 93% and 96% Re^{7+}, respectively.

Table S2. Overview of Re 4f fractions within the $AgReO_4$, 4Re-Ag and 4Re samples after oxidation, reduction, and re-oxidation treatment at 215 °C.

		Fraction of Re 4f (%)			
**Treatment**	**Sample**	**Re^{7+}**	**$Re^{2+ \text{ to } 6+}$**	**Re^{0}**	**Re^{total}**
**Oxidation**	$AgReO_4$	93	7	0	100
	4Re-Ag	92	8	0	100
	4Re	90	10	0	100
**Reduction**	$AgReO_4$	9	67	24	100
	4Re-Ag	22	76	2	100
	4Re	42	46	11	100
**Re-oxidation**	$AgReO_4$	93	7	0	100
	4Re-Ag	96	4	0	100
	4Re	68	32	1	100

Section B. Additional XRD data

Figure S3 shows the XRD of 4Re (Re/α-Al_2O_3). Only α-alumina peaks were detected, while ReO_x peaks were not. This means that the amount of Re_2O_7 present in this sample was either below the detection limit of the instrument, or it was not crystalline enough to be detected. Small diffraction peaks around 28, 37, 46 and 56 ° 2ϑ are satellite peaks of α-Al_2O_3 due to reflections by Co k_β which are not filtered 100%.

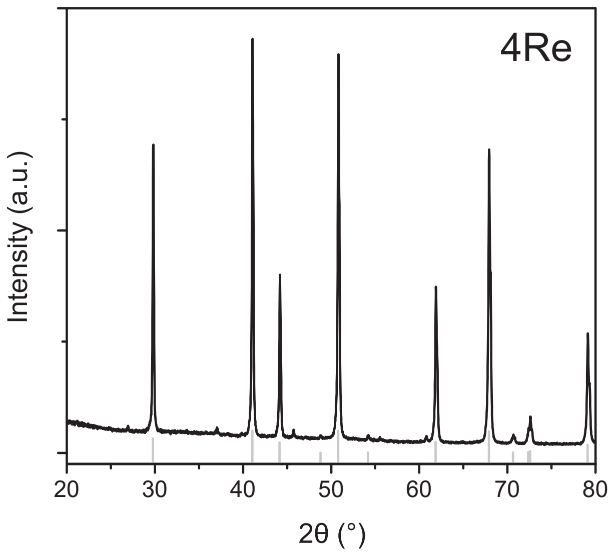

Figure S3. X-ray diffractogram of 4Re. The theoretical stick diffraction pattern of α-Al_2O_3 is shown below in grey.

After deposition of Ag on the Re-doped α-Al_2O_3 and varying the calcination time from 2 to 12 h, XRD and Rietveld refinements were performed using Bruker TOPAS software. The diffractograms of these samples as well as corresponding fits are shown in Figure S4. Diffractograms and fits of freshly prepared $AgReO_4$ on α-Al_2O_3 and after various treatments (catalysis, isomerization, chemisorption) are shown in Figures S5 and S6.

For all fits, a 3rd order Chebychev background and Lorentzian peak fitting were used. First α-alumina peaks were refined, followed by Ag, and then $AgReO_4$. When ReO_2 peaks were detected in the diffractogram, they were refined afterwards. Sample displacement was refined after fitting the various phases and fixed afterwards. Table S3 summarizes the results of all fits.

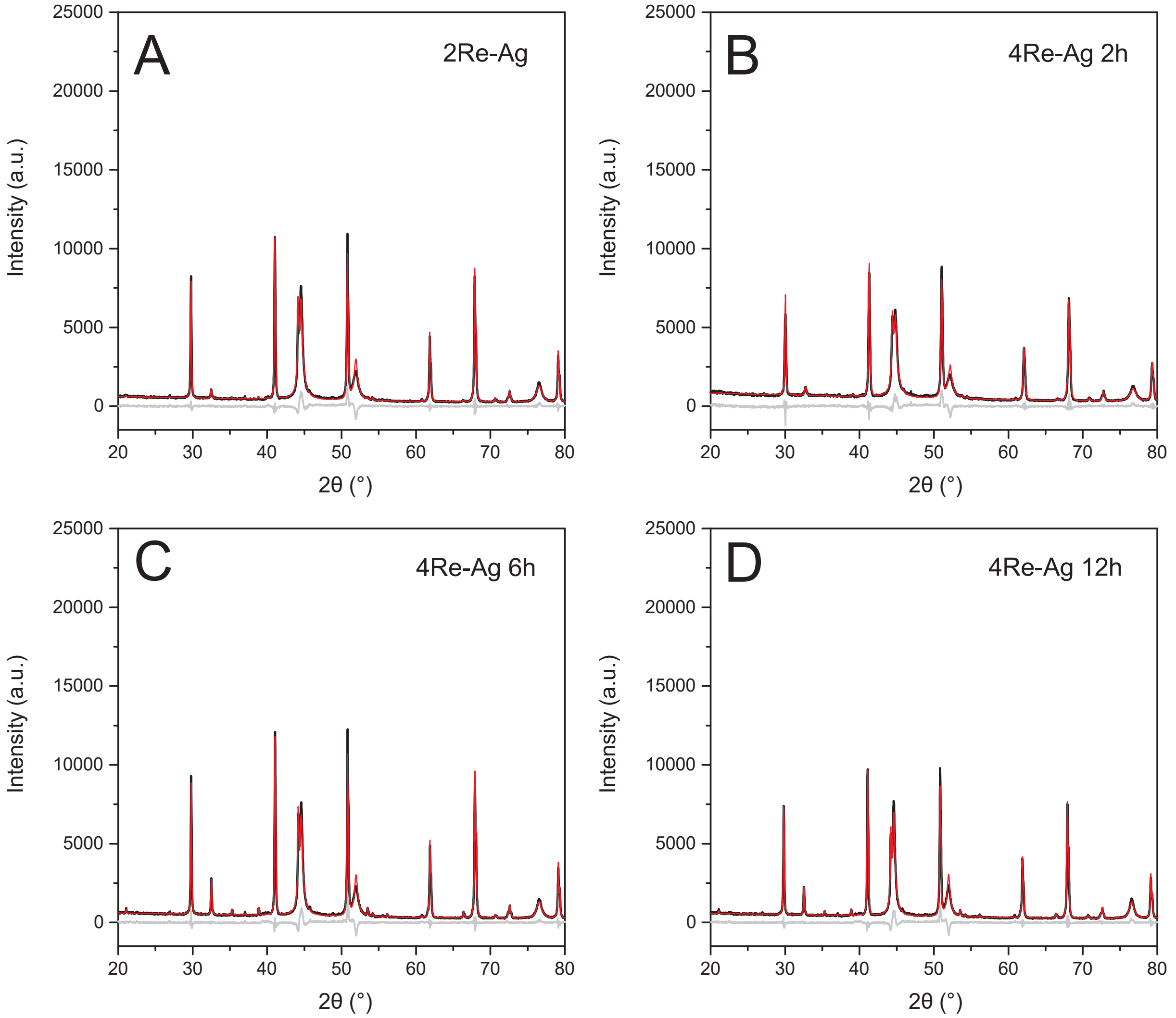

Figure S4. X-ray diffractograms (black) of Re-doped Ag catalysts with fits (red) and difference between the diffractogram and the fit (grey). 2Re-Ag is shown in (A), 4Re-Ag after 2 h calcination in (B), 4Re-Ag after 6 h calcination in (C) and 4Re-Ag after 12 h calcination in (D).

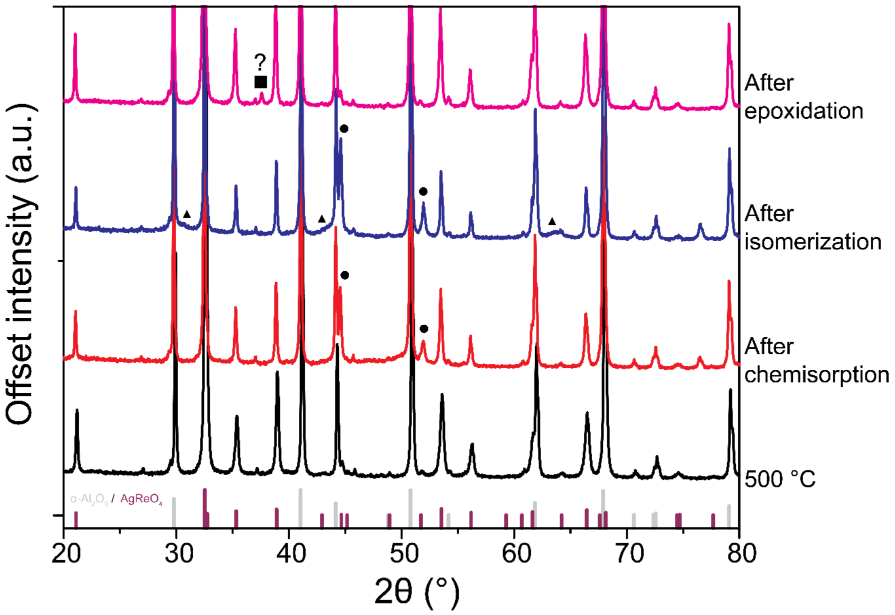

Figure S5. X-ray diffractograms of as prepared $AgReO_4$, after chemisorption, EO isomerization, and epoxidation. Theoretical stick diffraction patterns of α-Al_2O_3 and $AgReO_4$ are depicted below the diffractograms. Diffraction peaks of ReO_2 (▲) and Ag (●) are annotated accordingly. The diffraction peak after epoxidation around 38 ° *2ϑ* (■) is either AgO or AgCl.

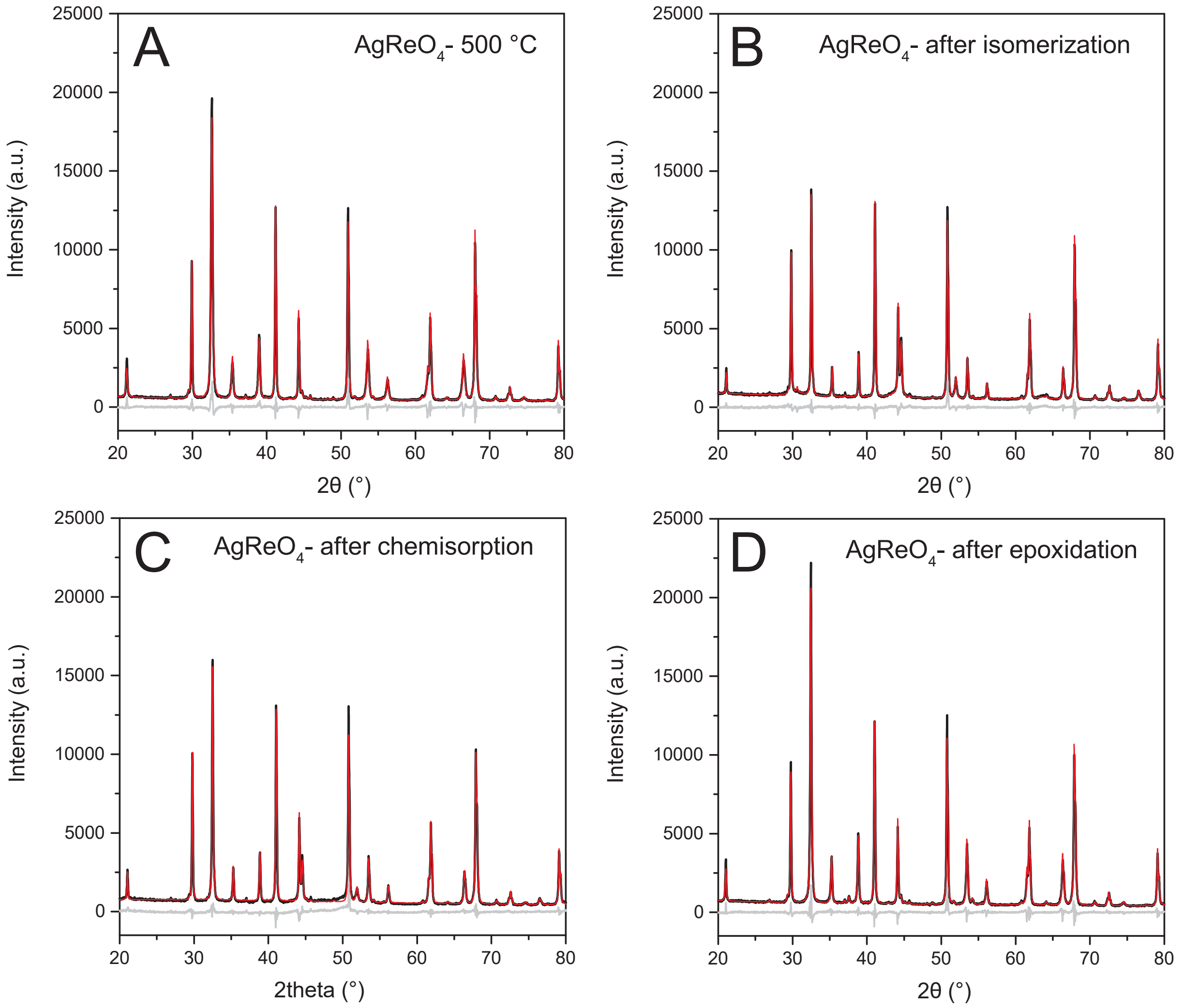

Figure S6. X-ray diffractograms (black) of $AgReO_4$ samples after various treatments with fits (red) and difference between the diffractogram and the fit (grey). The as prepared $AgReO_4$ sample after heat treatment at 500 °C (A), after EO isomerization (B), after O_2 chemisorption (C) and after ethylene epoxidation (D).

Table S3. Overview of crystalline phases present in the different Re-Ag and $AgReO_4$ samples based on XRD measurements.

Sample	α-Al_2O_3 (%)	Ag (%)	$AgReO_4$ (%)	ReO_2 (%)	R_{wp}
**2Re-Ag**	87.9	11.8	0.3	-	8.5
**4Re-Ag**					
**2h**	89.2	10.3	0.5	-	8.0
**After catalysis (EC)**	88.5	11.0	0.5	-	8.1
**After isomerization**	88.6	11.1	0.3	-	7.4
**After catalysis and isomerization (EC)**	88.6	10.9	0.5	-	7.6
**6h**	89.2	10.0	0.8	-	8.2
**12h**	88.4	10.8	0.8	-	7.9
**$AgReO_4$**					
**500 °C**	89.2	-	10.8	-	7.6

**After chemisorption**	92.1	1.3	6.6	-	8.2
**After isomerization**	91.7	1.9	5.0	1.4	5.5
**After catalysis (EC)**	89.9	-	10.1	-	7.0

Section C. O_2 chemisorption isotherms

O_2 chemisorption was performed with Re/α-Al_2O_3 and Re-Ag/α-Al_2O_3 samples. Isotherms are shown in Figure S7. For Re-Ag samples containing 2 and 4 at% Re, pressures up to 0.5 bar were needed to obtain saturation, compared to Ag and 0.4Re-Ag which saturated below 0.25 bar. The 2Re sample was also measured until 0.5 bar to confirm whether 0.25 or 0.5 bar was required.

O_2 chemisorption was also conducted with 80-90 mg of the Ag and 4Re-Ag catalysts after they had been stabilized in the ethylene epoxidation reaction for ca. 50 h with a maximum EC concentration of 1 ppm (Figure S7D). For these measurements, the used samples were evacuated for 30 min at 100 °C and 60 min at 215 °C prior to the O_2 analysis. Both samples showed an O_2 uptake of ca. 2.8 $\mu mol_{O2}\ g_{sample}^{-1}$. It seems that after catalysis the available Ag sites for both catalysts are similar. Repeating the measurement for the used 4Re-Ag with the typically used H_2 pretreatment (as described in Section 2.2 of the main text) resulted in an O_2 uptake of 69 $\mu mol_{O2}\ g_{sample}^{-1}$. Note that the O_2 uptake for the fresh 4Re-Ag catalyst was 75 $\mu mol_{O2}\ g_{sample}^{-1}$. After catalysis the O_2 uptake of the 4Re-Ag catalyst can almost be fully regained after a treatment in H_2.

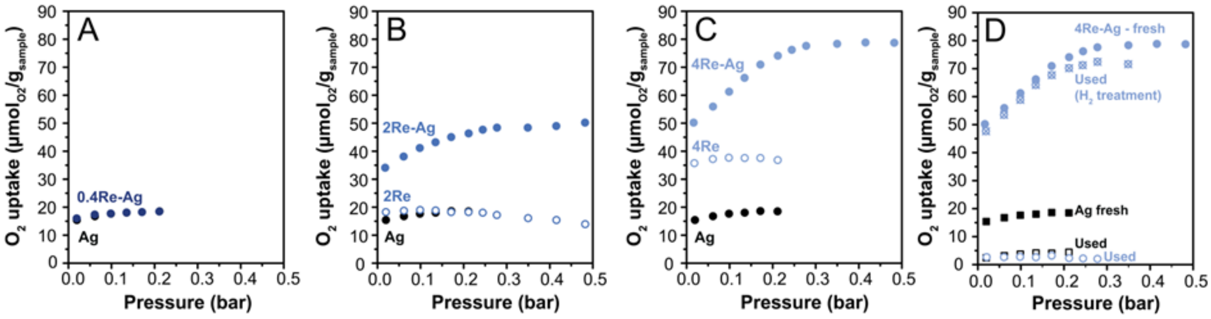

Figure S7. O_2 chemisorption isotherms of the Ag catalyst compared with 0.4Re-Ag (A), 2Re and 2Re-Ag (B), 4Re and 4Re-Ag (C), and Ag and 4Re-Ag used in ethylene epoxidation with ethyl chloride in the feed (D). For the used Ag catalyst no pretreatment was performed before the O_2 analysis. The used 4Re-Ag catalyst was first analyzed without pretreatment and afterwards with pretreatment in H_2 at 215 °C (following the earlier used protocol for all the fresh catalysts).

$AgReO_4/\alpha\text{-}Al_2O_3$ was also characterized with O_2 chemisorption. Figure S8 shows the effect of measuring these samples multiple times, as well as measuring with different equilibration times.

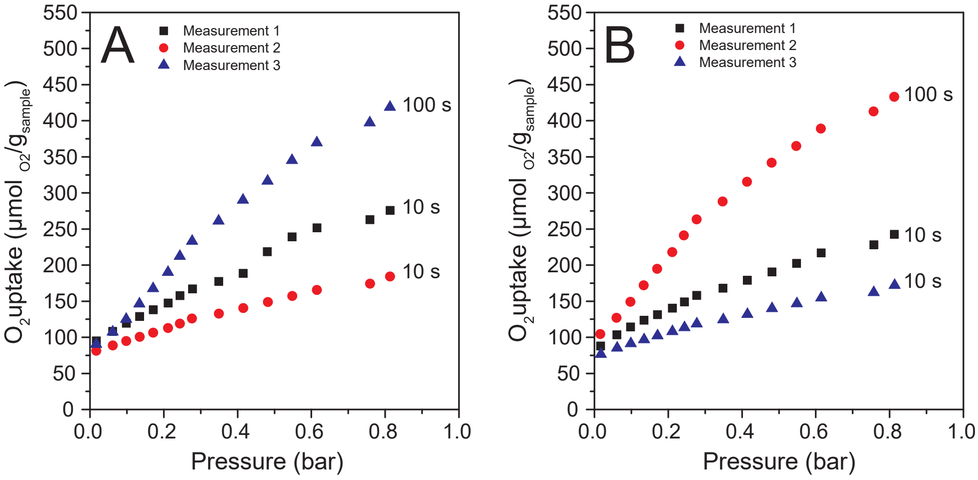

Figure S8. O_2 chemisorption isotherms of the $AgReO_4$ sample, studying the effect of equilibration time and order of measuring. In (A) the equilibration times were varied with first 10 s, another 10 s, and then 100 s. In (B) the order was 10 s, 100 s, and 10 s.

Section D. Additional catalytic data

Catalytic data of all the discussed catalysts are shown in Figure S9. Typically, 100 mg catalyst was tested with 500 mg SiC at 215 °C, with 7.5 vol% O_2, 7.5 vol% C_2H_4, 0-3 ppm EC in He. For 0.4Re-Ag, 2Re-Ag and 4Re-Ag the total gas flow was varied from 16-66 mL min^{-1}, whereas the Ag and $AgReO_4$ catalysts were only tested at 66 mL min^{-1}.

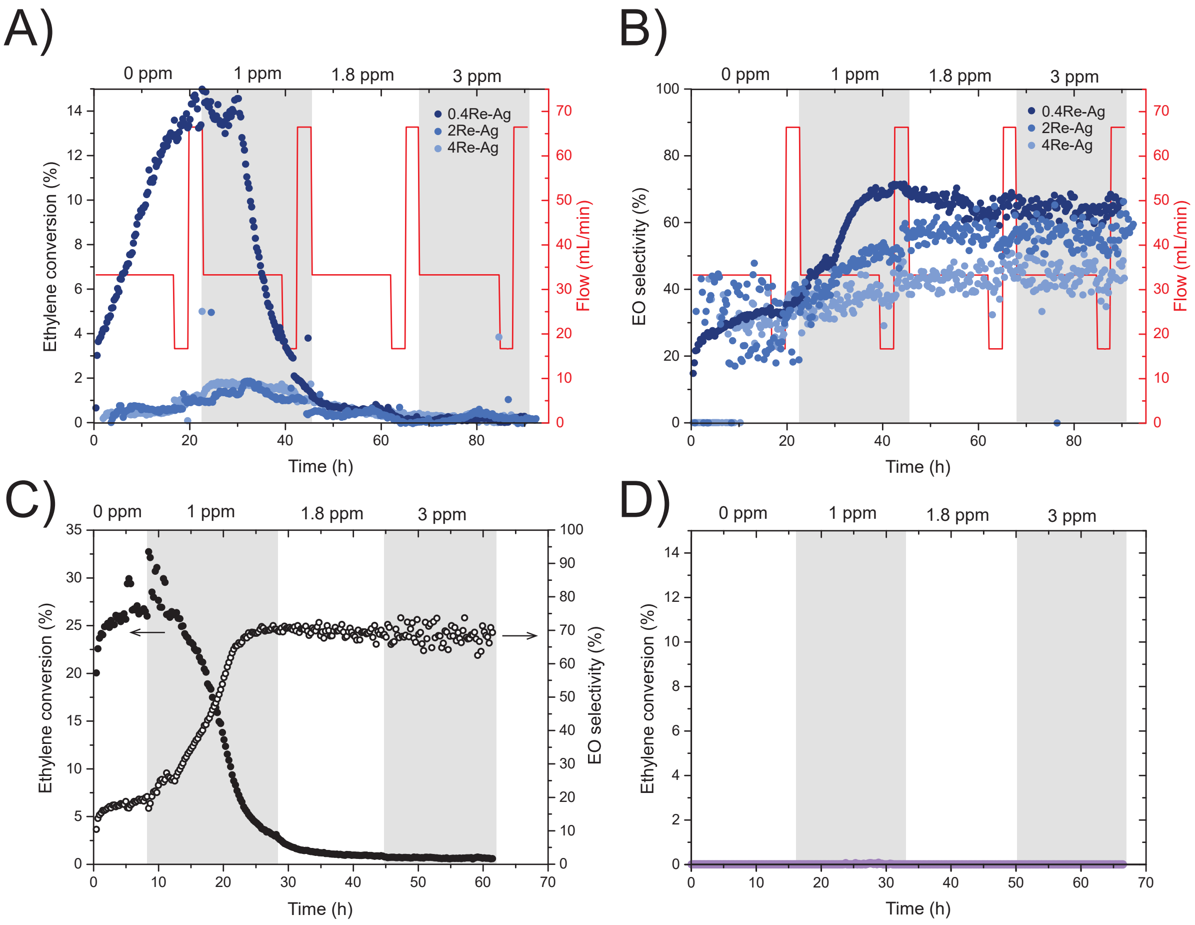

Figure S9. Catalytic data of 0.4Re-Ag, 2Re-Ag, 4Re-Ag (A and B), Ag (C) and $AgReO_4$ (D) at ethyl chloride (EC) concentrations between 0-3 ppm at 215 °C. Total gas flows were varied in A and B, and in C and D the total gas flow was kept at 66 mL min^{-1}.

To evaluate the effect of EC at lower concentrations, Ag and 4Re-Ag were tested with 0-1 ppm EC (Figure S10). For these tests, 100 mg catalyst was tested without SiC as these were also used for EO isomerization afterwards. The total gas flow was kept at 66 mL min^{-1}.

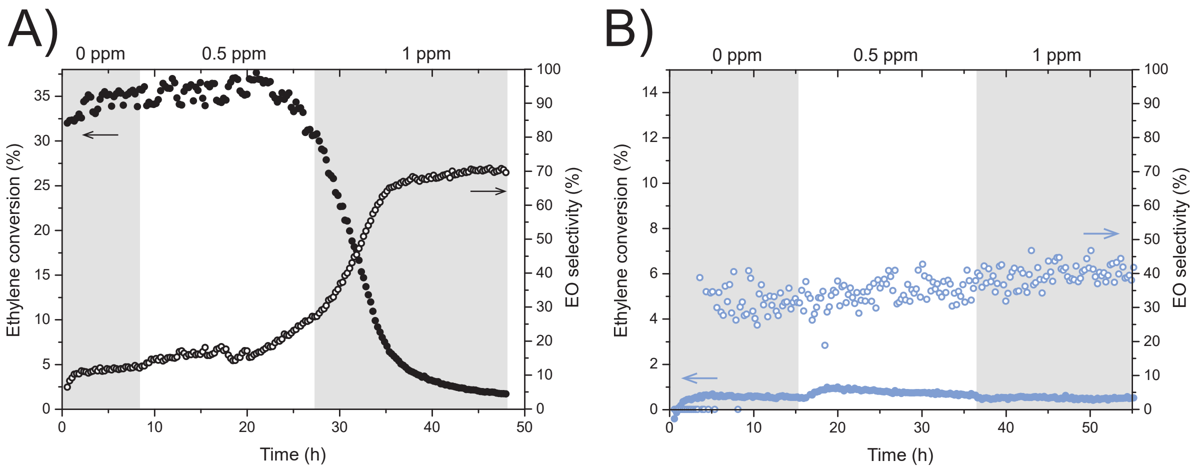

Figure S10. Catalytic tests of Ag (A) and 4Re-Ag (B) without SiC at 215 °C, with varying EC concentrations between 0-1 ppm.

Ag and 0.4Re-Ag catalysts were also tested with 0.25-1 ppm EC in the feed (Figure S11). Catalysts were diluted with SiC and tested at similar conditions as described earlier.

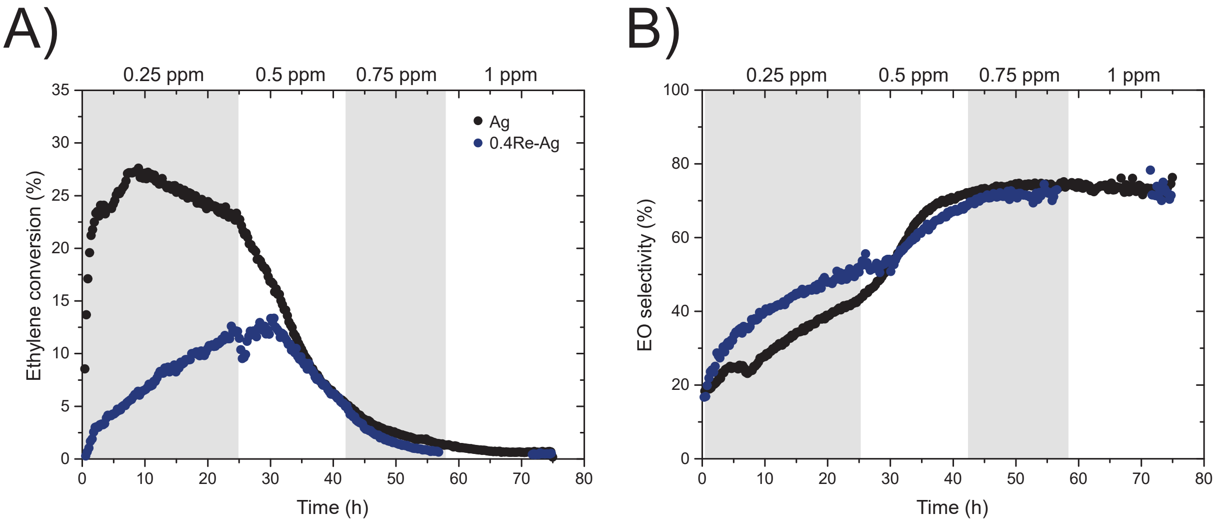

Figure S11. Ethylene conversion (A) and EO selectivity (B) of Ag (black) and 0.4Re-Ag (blue) at 215 °C with varying EC concentrations between 0.25-1 ppm.

Figure S12 summarizes all the catalytic data from Figures S9-S11, where ethylene conversion and EO selectivity is plotted as a function of EC concentration, in a total gas flow of 66 mL min^{-1}. Open symbols depict the tests without SiC in the feed.

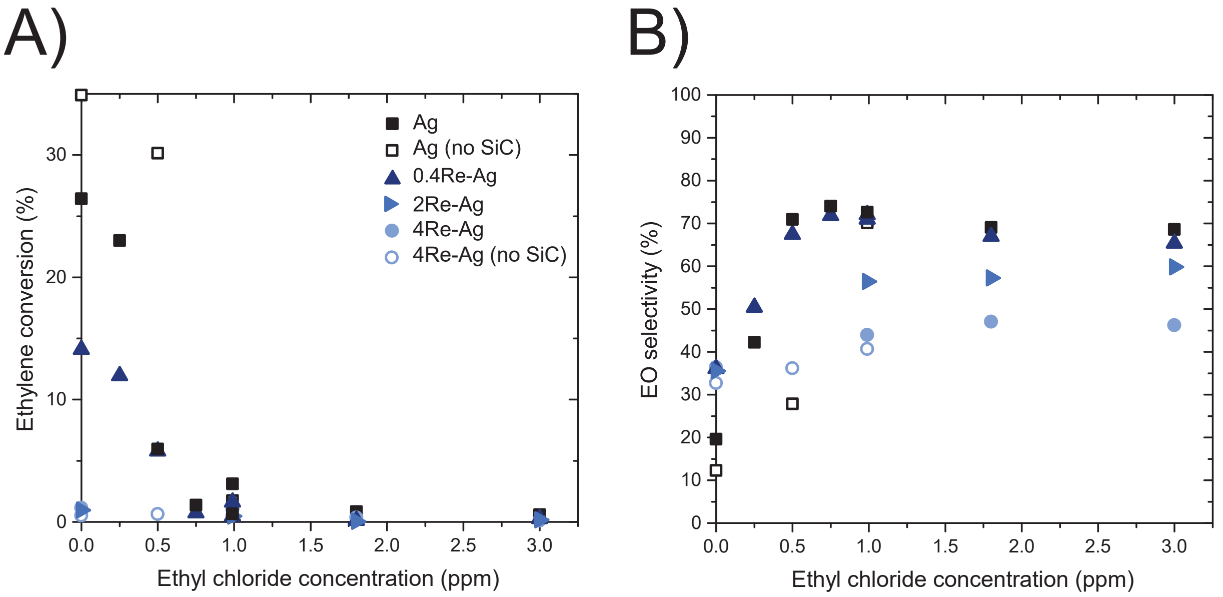

Figure S12. Overview of all catalytic tests with ethylene conversion versus EC concentration (A) and EO selectivity versus EC concentration (B). Open symbols depict tests without SiC.

Section E. Calculated equilibrium concentrations

Equilibrium concentrations of $AgReO_4$, Ag and ReO_x were calculated for the synthesis of Re-promoted Ag catalysts using HSC software [1]. Figure S13 shows the results of this calculation. It is clear that the formation of $AgReO_4$ is favored during calcination of Ag and Re_2O_7 species in an O_2-rich atmosphere.

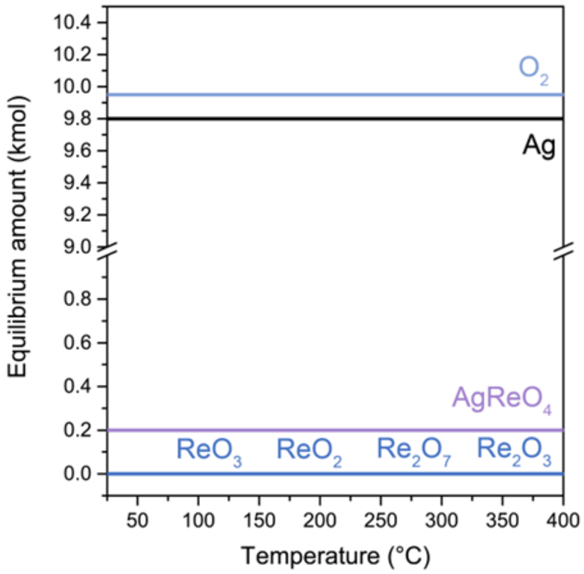

Figure S13. Equilibrium calculations using HSC for the system containing 10 kmol Ag, 0.1 kmol Re_2O_7 and 10 kmol O_2. This composition mimicks that of during calcination treatment of a sequentially impregnated Re-Ag catalyst. The results clearly show that the formation of $AgReO_4$ is thermodynamically favorable.

Equilibrium concentrations of $AgReO_4$ and Re_2O_7 were calculated for EO isomerization experiments without and with O_2 in the feed using HSC software [1]. Figure S14 shows the results of these calculations as a function of temperature. Starting concentrations were 10 kmol $AgReO_4$ or 10 kmol Re_2O_7, 1 kmol ethylene oxide, 0.5 kmol acetaldehyde, and for the experiment with oxygen 10 kmol O_2. Ag, ReO_2, Re_2O_7, Re_2O_3 and ReO_3 were selected as possible solid phases, and C_2H_4, CO_2, and H_2O as possible gas phases.

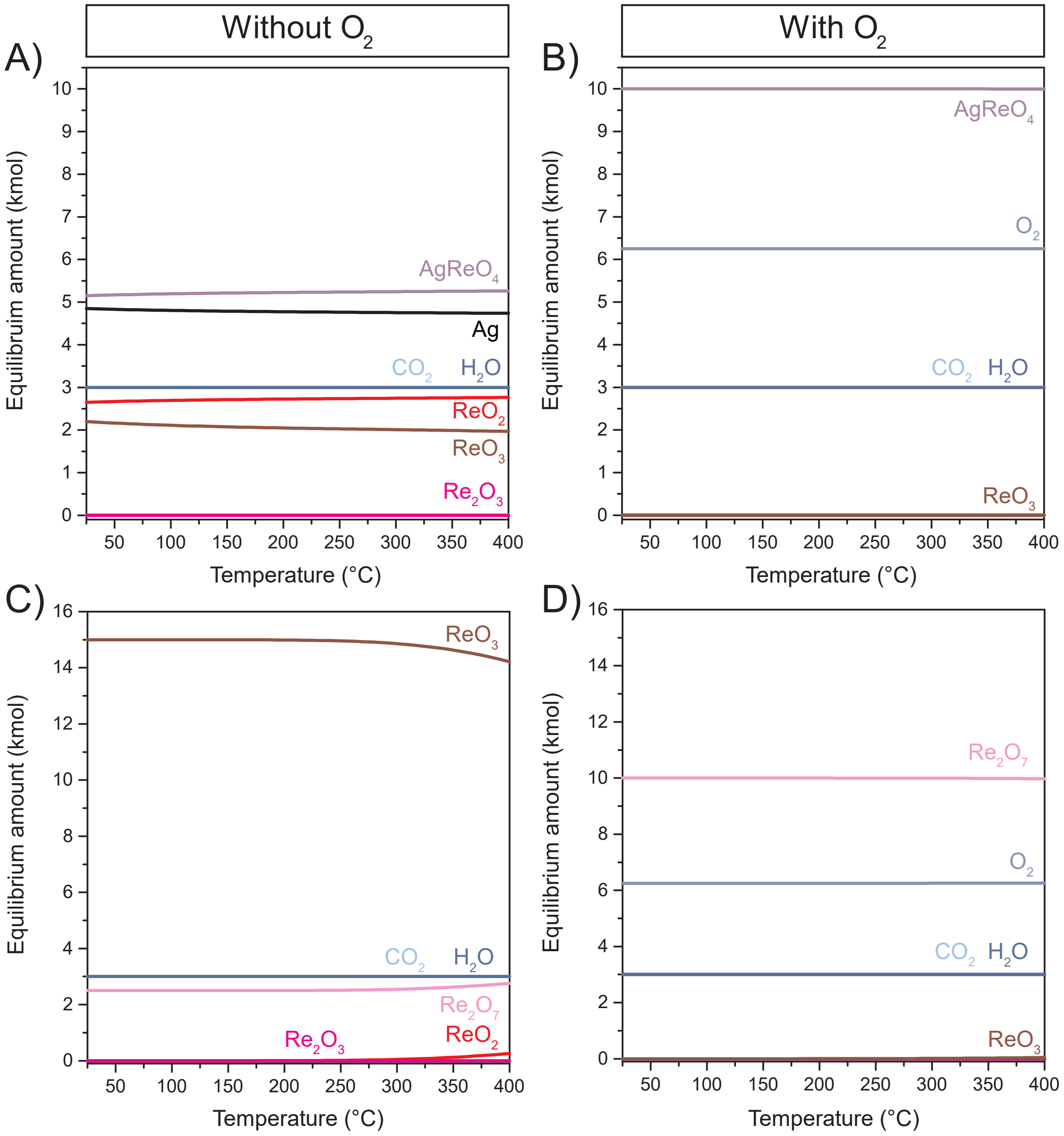

Figure S14. Equilibrium concentrations calculated with HSC for $AgReO_4$ in EO isomerization without O_2 (A) and with O_2 (B) in the feed, as well as for Re_2O_7 without O_2 (C) and with O_2 (D).

Without O_2 in the feed, $AgReO_4$ reduces to Ag, ReO_2 and ReO_3. Re_2O_7 reduces to ReO_3. With O_2 in the feed, $AgReO_4$ and Re_2O_7 are stable phases.

Section F. EO isomerization and stacked bed studies

During EO isomerization experiments without O_2 in the feed, ethylene was detected for the 4Re, 4Re-Ag and $AgReO_4$ samples. To evaluate if ethylene formed from ethylene oxide or acetaldehyde in the gas feed, stacked bed experiments were performed (Figure S15). In these experiments, the goal was to convert all the EO to acetaldehyde (AA). 100 mg of α-Al_2O_3 at 215-295 °C did not result in a full conversion of EO to AA, but 20 mg of γ-Al_2O_3 at 295 °C did (Frame A). The results of the stacked bed experiments at 295 °C were compared with EO isomerization experiments with the catalyst only (Frame B). Tests with Ag did not result in ethylene formation. $AgReO_4$ in stacked-bed experiments resulted in trace amounts of ethylene, compared to the catalyst-only experiment. Possibly, not all the EO was converted to AA, or $AgReO_4$ sample particles were present in the top part of the stacked bed.

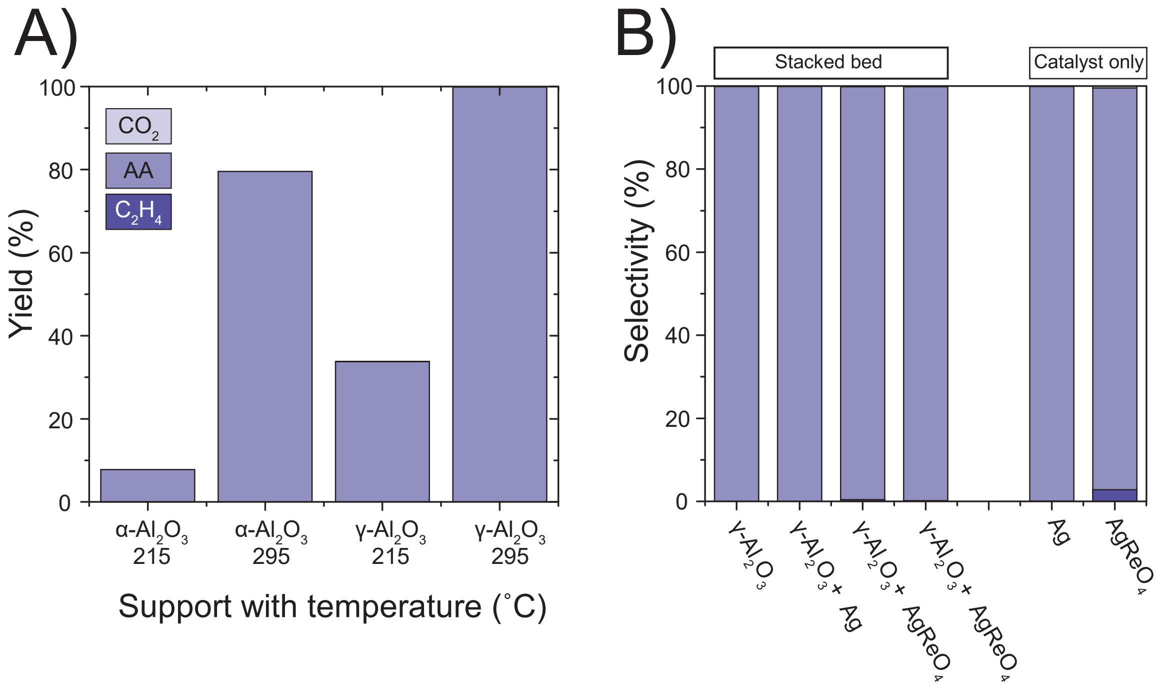

Figure S15. Acetaldehyde (AA) yields with different Al_2O_3 supports and temperatures (A), and comparing stacked-bed experiments with regular EO isomerization experiments at 295 °C (B).

The conversion of ethylene oxide to ethylene should result in the formation of O_2, and possibly also the formation of CO_2 as a result of combustion with ethylene (oxide) or acetaldehyde. Figure S16 shows the O_2 concentration and CO_2 selectivities of Ag, 4Re, 4Re-Ag and $AgReO_4$. For all samples, there is less O_2 in the feed compared to a test with an empty reactor. It is therefore difficult to quantify the amount of O_2 formed during isomerization. The samples can also chemisorb O_2 at the same temperature, which might underestimate the O_2 formation. Ag also shows a decreasing CO_2 selectivity over time, which suggests that the concentration of O_2 depletes over time. This might be explained by the desorption of weakly adsorbed oxygen from the surface.

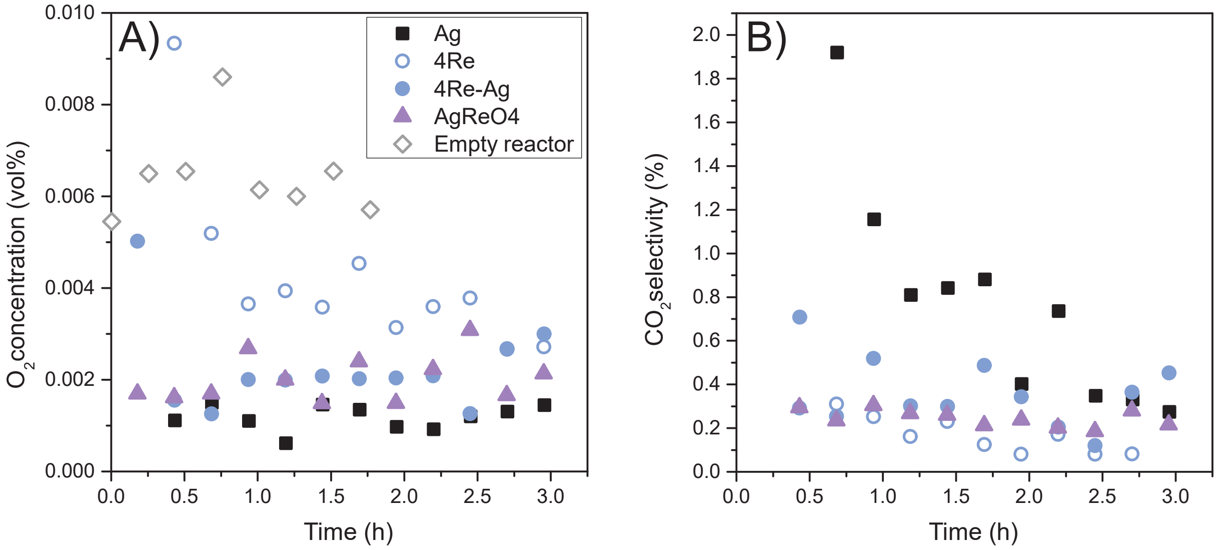

Figure S16. O_2 concentration during EO isomerization without O_2 in the inlet feed (A), and corresponding CO_2 selectivities (B).

After ethylene epoxidation/stabilization with EC in the feed, the Ag catalyst showed a decreased EO conversion as opposed to experiments without EC. To confirm this behavior, another 15 wt% Ag catalyst was tested for 50 h using similar reaction conditions (with EC) and afterwards tested in the EO isomerization reaction (Figure S17). This other catalyst was prepared using a different BASF Al-4196 α-alumina batch than before, and the silver precursor was decomposed with 100% O_2 instead of 25% O_2 in N_2. Ag particle sizes are 50 and 53 nm, respectively, determined with H_2 titration. Except for a higher EO selectivity during catalysis, which we ascribe to the different α-alumina batch, the EO conversion with EC is similar.

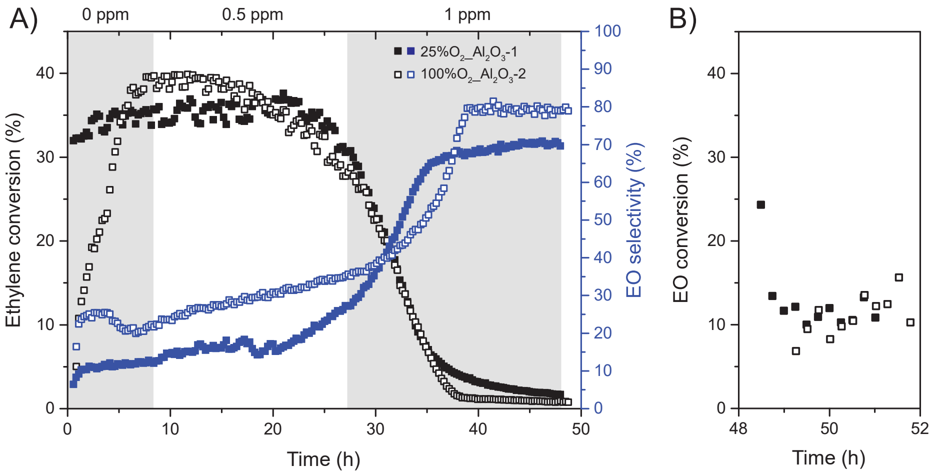

198 **Figure S17**. Catalysis data of two Ag catalysts decomposed with 25% or 100% O_2 and supported on
199 different batches of α-alumina (A) together with corresponding EO isomerization data with 1 ppm ethyl
200 chloride (B).

201

Section G. Additional SEM data of used catalysts

After ethylene epoxidation, the Re-promoted Ag catalysts were characterized with SEM to determine the surface averaged particle diameter of the used catalysts. Figure S18 shows SEM images of the used Ag, 0.4Re-Ag, 2Re-Ag and 4Re-Ag catalysts, together with the corresponding histograms of the fresh and used (stabilized) particle diameters. Particle diameters have increased from 70-80 nm to approximately 90 nm. This means that after ethylene epoxidation with EC in the feed, the Re promotion did not influence the Ag particle growth compared to the non-promoted Ag catalyst.

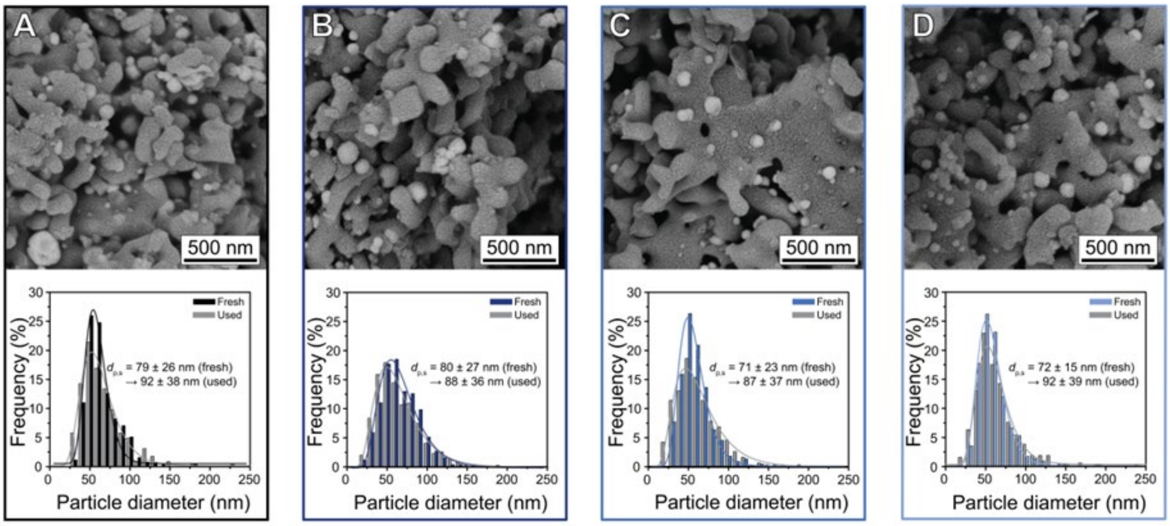

Figure S18. SEM images of the used Ag (A), 0.4Re-Ag (B), 2Re-Ag (C) and 4Re-Ag (D) catalysts with corresponding histograms of the fresh and used particle diameter distributions below. Catalysts after testing with EC during ethylene epoxidation for ca. 50 h. Surface averaged particle diameters ($d_{p,s}$) are also shown. For each sample, more than 200 Ag particles were measured.

References

[1] A. Roine, HSC Chemistry Software, (2023). www.metso.com/hsc.
